# High-yield charge separation along the alternative path in a photosynthetic reaction center: X-ray structure and electrostatic analysis

**DOI:** 10.1073/pnas.2609063123

**Published:** 2026-08-10

**Authors:** Stephen M. Keable, Rongmei Judy Wei, James C. Buhrmaster, Stephen Hippleheuser, Hiroki Makita, Philipp S. Simon, Gregory A. Tira, Moritz Kretzschmar, Isabel Bogacz, Kaitlyn M. Faries, Anthony Lan, Isabela I. Nangca, Miao Zhang, Margaret D. Doyle, Petko Chernev, Asmit Bhowmick, Daniel W. Paley, Nicholas K. Sauter, Aaron S. Brewster, Kensuke Tono, Shigeki Owada, Junko Yano, Vittal K. Yachandra, M. R. Gunner, Christine Kirmaier, Dewey Holten, Deborah K. Hanson, Philip D. Laible, Jan F. Kern

**Affiliations:** ^a^ Molecular Biophysics and Integrated Bioimaging Division, Lawrence Berkeley National Laboratory, Berkeley, CA 94720; ^b^ Ph.D. Program in Chemistry, The Graduate Center, City University of New York, New York, NY 10016; ^c^ Department of Physics, Division of Sciences, City College of New York, New York, NY 10031; ^d^ Biosciences Division, Argonne National Laboratory, Lemont, IL 60439; ^e^ Department of Biology, Humboldt-Universität zu Berlin, Berlin DE-10115, Germany; ^f^Department of Chemistry, Washington University, St. Louis, MO 63130; ^g^ Molecular Biomimetics, Department of Chemistry—Ångström, Uppsala University, Uppsala, Sweden; ^h^RIKEN SPring-8 Center, Sayo-cho, Sayo-gun, Hyogo 679-5148, Japan; ^i^ Japan Synchrotron Radiation Research Institute, Sayo-cho, Sayo-gun, Hyogo 679-5198, Japan; ^j^ Ph.D. Program in Physics, The Graduate Center of the City University of New York, New York, NY 10016

**Keywords:** photosynthesis, electron transfer, purple bacterial reaction center

## Abstract

In all photosynthetic reaction centers (RC), transmembrane electron-transfer (ET) cofactor pathways are coordinated by homologous peptides and arranged with approximate C_2_ symmetry, providing two possible paths for charge separation (designated A and B). In type II RCs—Photosystem II and the purple bacterial RCs—only the A branch is active for ET. A variant bacterial RC containing nine amino acid substitutions that result in high-yield ET along the normally nonfunctional B branch was designed. Structural and theoretical studies were combined to understand factors that control unique ET reactions in the complex. Serial femtosecond crystallography performed at an X-ray Free Electron Laser afforded the room temperature X-ray-damage-free crystal structure of the variant RC, revealing interactions between the substituted amino acids and the ET cofactors. The positions of the bacteriochlorin cofactors on the symmetry-related pathways are unaffected, underscoring that the substitutions alter the energetics of ET, leading to the functional changes. In the binding pockets for the terminal electron acceptor quinones, alterations in the positions of neighboring amino acids and water molecules are coordinated with the change of the substituted side chains. Modifications deactivated A-branch ET and activated transmembrane charge separation along the B branch. Electrostatic calculations based on the structure reveal how differences in stabilization of charge-separated states by the protein environment underlie the change in direction of electron flow in this variant RC and provide insights into mechanisms by which B-branch charge separation across the photosynthetic membrane can be achieved in high yield.

## Significance

Bacterial reaction centers are transmembrane protein–cofactor complexes that are central to an ancient and preserved photosynthetic process. They harvest light energy to drive electron transfer across a membrane through a series of redox-active cofactors in symmetry-related pathways (designated A and B). Only the A branch operates and the amino acid environment around the cofactors tunes them for reactivity. A series of substitutions was found to inactivate the A pathway and direct electron flow to the B pathway instead. X-ray Free Electron Laser crystallography produced a structure of this complex which, along with structure-guided electrostatic calculations, allows for a better understanding of how these substitutions tune the electron transfer pathways. Such insights are important for protein design approaches and biomimetic applications.

**Fig. 1.**
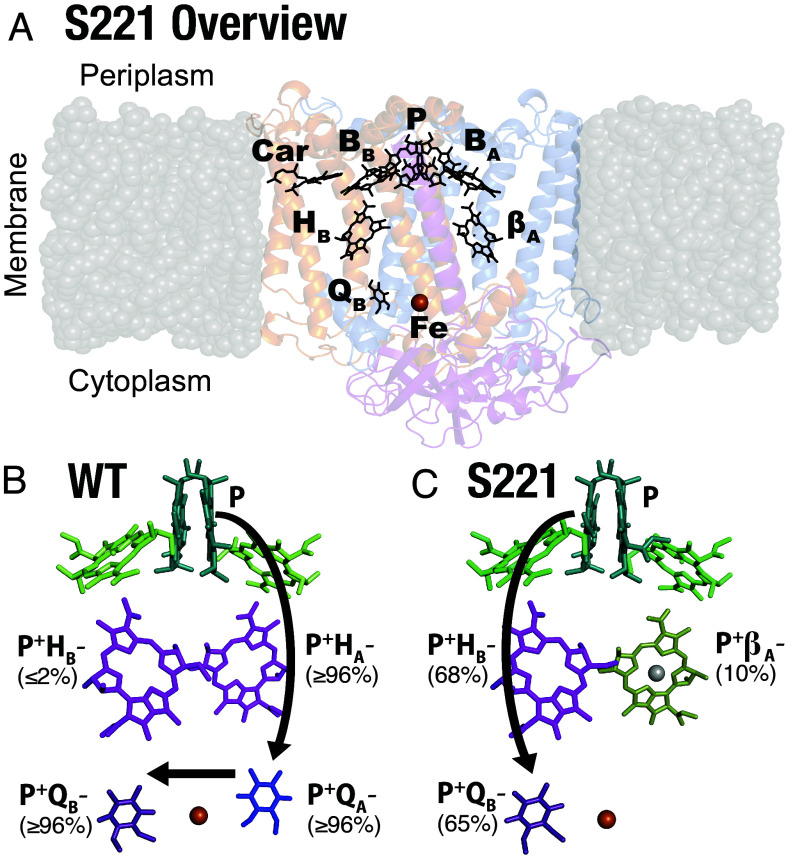
Structure of and electron transfer in the purple bacterial reaction center. (*A*) The structure of the reaction center with the position of the membrane indicated and the three protein subunits shown in blue (L), gold (M), and purple (H). Electron transfer cofactors for the S221 variant are shown in black and the nonheme Fe as orange sphere. (*B* and *C*) Electron transfer cofactors. The primary donor BChls P_A_/P_B_ are shown in teal on the *Top*, followed by BChls B_A_ and B_B_ (green) and the BPhs H_A_ and H_B_ (magenta) in the WT or the BPh H_B_ and a BChl molecule (β_A_, olive) in the S221 structure. On the acceptor side, the ET is completed by two UQ molecules (Q_A_ and Q_B,_ blue and purple) in the WT RC, whereas in the S221 RC only one UQ molecule is present (Q_B_, purple). Percent yields of charge-separated states (5) are given, e.g., P^+^Q_B_^−^, with ET from P to Q_B_ via the A branch in the WT (panel *B*) vs. via the B branch in the S221 variant RC (panel *C*). The remaining 22% of P* in the S221 variant decays by internal conversion to the ground state. The carotenoid molecule (panel *B* and *C*) and phytyl tails of the cofactors have been removed from the figure for visual clarity.

The ability to convert light energy into chemical energy was a pivotal inflection point in the history of life on earth. The understanding of how these evolved proteins function is of great interest to researchers as they provided clues for optimization of photosynthesis and development of bioinspired artificial photosynthetic systems. All photosynthetic reaction centers (RCs) are able to generate long-lived charge-separated states that allow for electron transfer (ET) events with near unity quantum yield that utilize a structure featuring two symmetry-related branches of ET cofactors (1–4). The purple bacterial RCs (PBRC) contain a dimer (P) consisting of a pair of bacteriochlorophyll (BChl) molecules (P_A_ and P_B_) on the periplasmic side of the complex, each located next to a single BChl (B_A_ and B_B_) then followed by a bacteriopheophytin (BPh) molecule (H_A_ and H_B_) arranged with C_2_ symmetry (Fig. 1 *A* and *B*). Interestingly, light-driven ET in the wild type (WT) proceeds from the excited primary electron donor P* along only one (the A branch) of the two branches to the first quinone acceptor (Q_A_), a ubiquinone-10 (UQ) molecule close to the cytoplasmic side. In the last step of the process, an electron is transferred from Q_A_ to the symmetry-related ubiquinone-10 (Q_B_) on the B branch (Fig. 1*B*). While all other cofactors are fixed in the protein, the final acceptor Q_B_ accepts two electrons and two protons in two turnovers and leaves the PBRC as Q_B_H_2_.

All cofactors are coordinated by the two subunits L and M that form a heterodimer along the same C_2_ pseudo-symmetry axis that applies to the two cofactor branches. The protein complex is completed by the third subunit, called H. While the general architecture of the branches is highly symmetrical, there are several deviations in the amino acid environment of the symmetric cofactors, leading to clear differences in the properties of the A- and B-branch cofactors, affecting their redox potentials and the electronic coupling between them. These asymmetries result in highly optimized ET along the A branch whereas ET along the B branch is suppressed in the WT (Fig. 1*B*). Understanding how this charge separation process works in nature with such high efficiency and specificity is important for using this process to harness light energy in artificial photosynthetic systems.

Over the years, in an effort to understand the reasons for the unidirectionality of ET, a multitude of individual and combined site-directed amino acid substitutions have been made in PBRCs to direct ET to the B branch (5–11). Substitutions near the bacteriochlorin cofactors (P_A_, P_B_, B_A_, B_B_, H_A_, and H_B_) have been made to alter their redox properties and thereby the free energies of the associated charge-separated states (e.g., P^+^B_A_^−^ and P^+^B_B_^−^), near the quinones (Q_A_ and Q_B_) to alter redox properties and binding, and in between to enhance intercofactor electronic coupling. The goal has been to decrease the rate and yield of one or more of the ET steps on the A branch and to enhance the corresponding processes on the B branch (Fig. 1*C*). All of these features are combined via nine amino acid substitutions in a site-directed mutant of the *Cereibacter* (*C.*; formerly *Rhodobacter*) *sphaeroides* RC, designated “S221,” that was recently engineered and characterized (5). The nine substitutions and their purposes are given in Table 1. Spectroscopic measurements on the S221 RC showed that the yield of B-branch primary ET (P* → P^+^B_B_^−^ → P^+^H_B_^−^) is 68% and the yield of secondary ET (P^+^H_B_^−^ → P^+^Q_B_^−^) is 95% resulting in an overall yield of P^+^Q_B_^−^ of 65% (0.95*0.68). The yield of P* decay to the A-side cofactors is reduced to 10% and the remaining 22% of P* decays by internal conversion to the ground state.

**Table 1.** Substitutions in the S221 mutant RC and their purposes

Site	Substitution	Purpose
L-181	F → Y	Stabilize P^+^B_B_^−^; Enable B-Side ET^*^
L-185	L → W	Stabilize P^+^H_B_^−^; Enable B-Side ET^†^
L-193	L → T	Promote Q_B_ binding; Enable B-Side ET^‡^
L-216	F → W	Promote Q_B_ binding; Enable B-Side ET^‡^
M-133	T → E	Stabilize P^+^H_B_^−^; Enable B-Side ET^§^
M-210	Y → F	Destabilize P^+^B_A_^−^; Disable A-Side ET^§^
M-214	L → H	Replace H_A_ with BChl (β_A_); Disable A-Side ET^¶^
M-252	W → V	Blocks binding of Q_A_; Eliminate Q_A_ activity^#^
M-260	A → W	Blocks binding of Q_A_; Eliminate Q_A_ activity^#^

^*^Calculations (12–14) indicate that (native) Tyr M-210 stabilizes P^+^B_A_^−^ by ~200 meV whereas (native) Phe L-181 does not provide such stabilization of P^+^B_B_^−^.

^†^The aromatic Trp is employed to stabilize nearby charge (i.e., H_B_^−^), just as Trp residues facilitate hole/electron transfer in other proteins (15, 16).

^‡^Formation of a hydrogen bond between Thr L-193 and Trp L-216 positions the Trp for π-π interactions with Q_B_ (5).

^§^A hydrogen bond between Glu M-133 and the ring V keto group of H_B_ is indicated by the red shift of the Q_x_ band of H_B_ from 528 to 535 nm (17). This hydrogen bond should stabilize P^+^H_B_^−^ by 50 to 100 meV based on the effects of hydrogen bonds to BChl *a* and BPh *a* (18, 19).

^¶^The pigment substitution (6, 20–23) spectrally isolates the Q_x_ band of H_B_ from that of β_A_ and makes spectral diagnosis of P^+^H_B_^−^ unambiguous. P^+^β_A_^−^ is expected to lie higher in free energy than P^+^H_A_^−^ by 150 to 300 meV based on the difference in reduction potentials of BChl *a* and BPh *a* in vitro (24, 25).

^#^The M-W252V (26, 27) and M-A260W (28) substitutions weaken binding of Q_A_ resulting in its displacement or absence. Q_A_ is absent in the X-ray crystal structures of the M-W252V (PDB ID: 7MHA) (26) and M-A260W (PDB ID: 1QOV) RCs of *C. sphaeroides* (29).

Knowledge about the structural consequences of amino acid substitutions has been generally limited to early studies on select mutant RCs with relatively low B-branch activity (10, 26, 30). The opportunity now arises to determine the structure of an PBRC with high B-branch activity. Using femtosecond X-ray Free Electron Laser (XFEL) crystallography, one can collect structural data at room temperature before the onset of X-ray induced radiation damage (31–34). This method provides a way to observe the structural consequences of site-directed substitutions at nearly physiological temperature conditions.

The cofactor arrangement in the room temperature XFEL crystal structure of the *C. sphaeroides* S221 variant RC that “re-wires” ET from the normally active A branch to the normally inactive B branch is shown in Fig. 1*C*. The structure reveals the nine amino acid substitutions with no posttranslational modifications. The positions of the six bacteriochlorin cofactors are unchanged compared to the WT RC, validating the design of the substitutions to primarily affect the energetics of the ET processes. Some notable structural effects were observed in the quinone pockets that are potentially important for function. The availability of detailed structural information for the S221 RC also allowed us to perform calculations of the midpoint potentials of the cofactors, using the Multi-Conformational Continuum Electrostatic (MCCE) method (35). Comparison of the calculated midpoint potentials for the cofactors in the S221 mutant RC structure with those calculated for the WT structure supports and rationalizes the observed B-branch ET in this variant. Collectively, the structural findings and results of electrostatic calculations presented herein and prior spectral and kinetic studies (5) on the *C. sphaeroides* S221 mutant RC give important insights into the molecular mechanisms of the charge-separation process in PBRCs. These results have the potential to help design photosynthetic RCs by tuning the cofactor properties, the rates and yields of ET, and the optimization of photochemical systems for solar-energy conversion. In addition, elucidating details about fine-tuning of cofactor properties by the protein environment can provide important insights for protein design where, for example, targeted modifications of existing enzymes are intended to achieve new functionality.

## Results

### Structural Consequences of the Substitutions.

The S221 mutant RC with nine amino acid substitutions is incapable of supporting photosynthetic growth of the organism. Its expression level in dark-grown cultures is relatively high at ~62% of that of the WT RC and is markedly improved over that of other earlier mutant RCs where replacing one or more of the residues substituted in the S221 RC with the native residue led to reduced RC expression (*SI Appendix*, Fig. S1). In one example, the expression level of an RC with only six of the nine substitutions is only ~12% of that of the WT RC. In addition to the high expression level, the multiply mutated protein is very stable during purification and crystallization, allowing for the generation of sufficient amounts of microcrystals for subsequent XFEL crystallography.

**Fig. 2.**
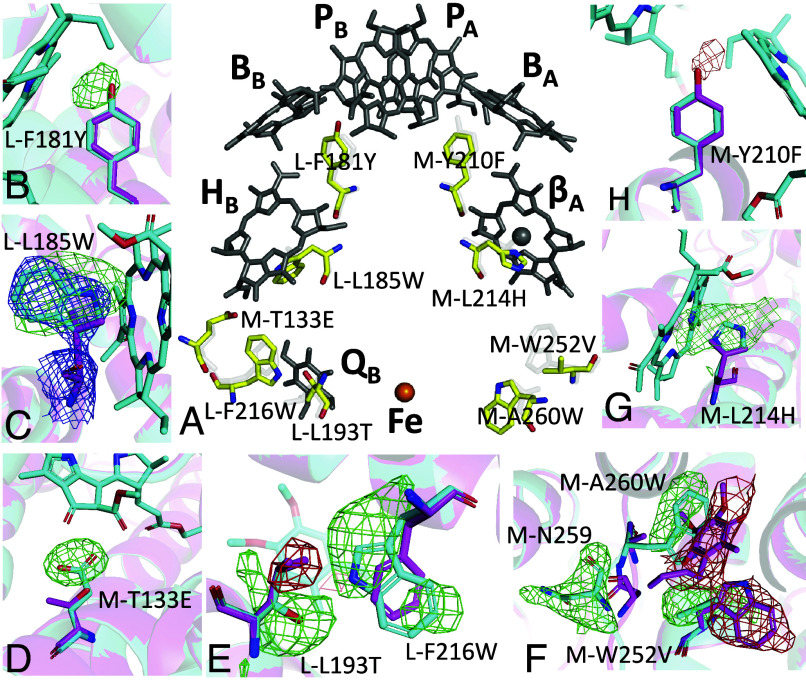
Overview of substitutions and resulting electron density changes in the S221 RC. Nine site-directed amino acid substitutions were introduced to drive ET along the B-branch pathway. (*A*) Redox-active cofactors are dark gray, substitutions are yellow, and pale gray shadows are native amino acids. (*B*–*H*) Red and green F_o_-F_c_ difference electron density contoured to 3.0σ confirmed the presence of substituted residues with the S221 variant structure (cyan) superimposed on the WT structure (PDB ID: 2J8C; magenta). Blue 2F_o_-F_c_ electron density contoured to 1.5σ is shown to illustrate the unexpected edge-to-face T-stacked orientation of the L-W185 residue with regard to H_B_ (*C*). Unexpected conformations of neighboring residues are also seen in the Q_A_ pocket (*F*). In the view shown here (panel *A*), the macrocycle of P on the right side (near B_A_) and with its central Mg coordinated by His L-173 is P_A_ and the macrocycle of P on the left side (near B_B_) and with its central Mg coordinated by His M-202 is P_B_. Q_A_ is absent and BPh H_A_ in the WT structure is replaced by BChl, denoted β_A_ in the S221 RC structure (*G*).

The structure of the S221 variant RC was obtained at room temperature using serial femtosecond crystallography at 2.70 Å resolution with R/R_free_ values of 21.5/23.3 (Table 2 and *Materials and Methods*). The overall secondary structure of the S221 variant was compared to the WT RC structure (PDB ID: 2J8C) (36) using the protein structure comparison service SSM at the European Bioinformatics Institute (http://www.ebi.ac.uk/msd-srv/ssm). Examination of the RMSD among all residues reveals the close similarity of the structures, with an average RMSD of 0.4 Å. Electron density difference maps (F_o_-F_c_) computed as the difference between the observed structure factors (F_o_) and the calculated structure factors for the WT structure (F_c_) provided a straightforward path to model in the nine site-directed amino acid substitutions (Fig. 2) that were also confirmed with unambiguous 2mF_o_-DF_c_ electron density maps after refinement. There are no significant changes in the positions/structures of the six bacteriochlorin cofactors or other structural changes (other than the amino acid side-chain replacements). However, there are conformational changes near the quinones that are of functional consequence.

**Table 2.** Statistics for crystallographic data collection and structure refinement for the S221 variant RC

Beam Line	SACLA BL3-EH2
Resolution range refined (Å)^*^	44.84 to 2.70 (2.75 to 2.70)
Wavelength (Å)	1.241
Space group	P 21 21 21
Unit cell parameters (Å)	a = 142.05 (+/−) 0.22 b = 139.64 (+/−) 0.47 c = 77.44 (+/−) 0.13
Lattices merged	4,252
Unique reflections^*^	42,976 (2,074)
Completeness^*^	99.77% (97.33%)
CC_1/2^*^_	97.2% (14.3%)
Multiplicity^*^	48.52 (7.2)
I/σ(I)^*^,^†^	2.82 (0.27)
Wilson B-factor (Å^2^)	79.02
R-factor/ R-free	21.47%/23.33%
Nonhydrogen atoms	7,155
Macromolecule atoms	6,515
Ligand atoms	594
Solvent atoms	46
Protein residues	828
RMS bonds (Å)/angles(deg)	0.004/0.67
Ramachandran favored/outliers	96.59%/0.12%
Clashscore	5.77
Average B-factor (Å^2^)	83.59

^*^Numbers in parentheses are for upper resolution bin.

^†^As described in ref. 37.

### P_A_/P_B_ and B_A_/B_B_ Region.

**Fig. 3.**
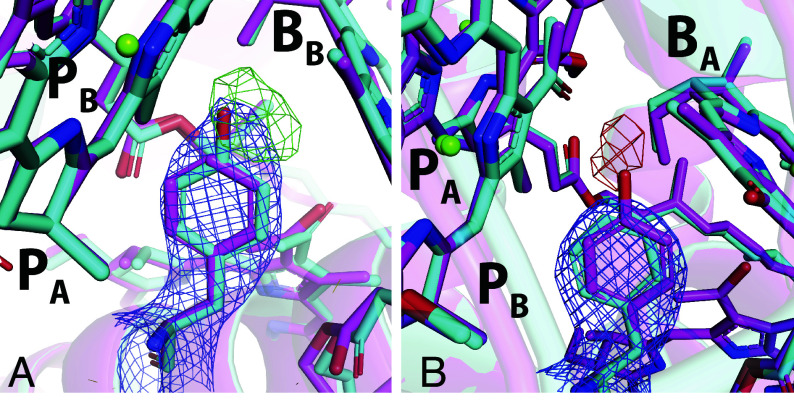
Structural consequences of a swap of Phe and Tyr, conserved asymmetric residues near P (YF swap). BChl molecules P and B on both branches are shown together with the L-181 and M-210 residues. Structure of the S221 variant RC (cyan) superimposed with WT (PDB ID: 2J8C; magenta) with F_o_-F_c_ difference electron density shown in red and green contoured to ±3.0σ, and 2F_o_-F_c_ density shown in blue, contoured to 1.5σ. (*A*) The area around residue L-181 (B branch) shows positive F_o_-F_c_ density (green) for the newly introduced OH group confirming the L-F181Y substitution. (*B*) The area around residue M-210 (A branch) shows negative F_o_-F_c_ density (red) indicating the absence of the tyrosine OH group, confirming the M-Y210F substitution. The axial ligands to P_A_ and P_B_ are His L-173 and His M-202, respectively.

The aromatic residues M-Y210 and L-F181 are in symmetry-related positions along the two branches near B_A_ and B_B_, respectively, and the keto (C=O) group of the ring I acetyl substituent of P_A_/P_B_. The electron density difference map peaks clearly indicate that amino acids M-Y210 and L-F181 have been substituted with a phenylalanine and tyrosine, respectively (Fig. 3). Other than the addition or subtraction of the hydroxyl group, no major structural differences were observed relative to the previously published high-resolution WT structure (PDB ID: 2J8C) (36).

**Fig. 4.**
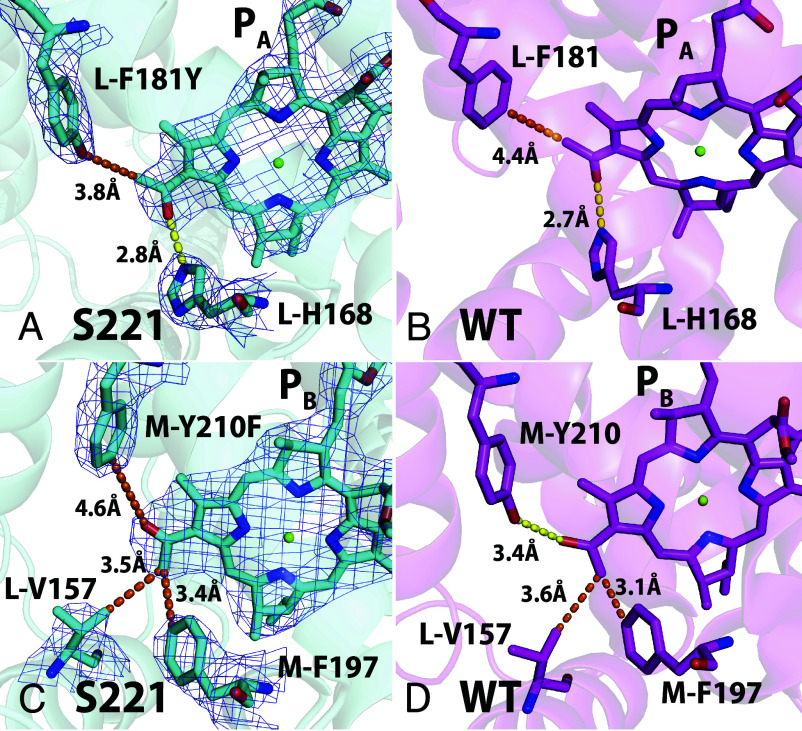
The YF substitutions near the ring I keto groups of P. The L-F181Y and M-Y210F substitutions lie in close proximity to the ring I keto groups of the P_A_ and P_B_ BChls. The S221 variant RC is shown in cyan and the WT (PDB ID: 2J8C) is shown in magenta with 2F_o_-F_c_ electron density maps contoured to 1.5σ (blue mesh). (*A*) The tyrosine hydroxyl group of L-F181Y is beyond hydrogen bonding distance to the keto group of P_A_, with L-H168 still within coordination distance. (*B*) In the WT RC, L-H168 is potentially hydrogen bonded to the keto group of P_A._ (*C* and *D*) On the B branch, the M-Y210F substitution removes the potential hydrogen-bond interaction with the ring I keto group of P_B_ in the WT (*D*) and no other H-bond interaction can be formed. Distances are represented as orange dashed lines, H-bonds are represented as yellow dashed lines.

The “YF swap” of M-Y210F/L-F181Y can modulate the interactions between the BChl cofactors and nearby amino acids. The S221 RC structure does not show any obvious hydrogen bonding partner for the newly introduced Tyr phenolic group at L-181 (Fig. 4*A*). In WT RCs, the ring I keto group of P_A_ forms a hydrogen bond with His L-168 (Fig. 4*B*) (18). At the current resolution of the S221 RC structure, modeling of the orientation of the ring I keto group of the BChl and BPhe cofactors is challenging and we relied on the orientation observed in previous high-resolution models for the WT, except for cases where a clear hydrogen bonding interaction was visible.

For the P_A_ keto group it is not obvious if it points toward L-168 or the inserted OH group of L-Y181, but the distance between it and His L-168 has elongated slightly from 2.7 to 2.8 Å. Upon modeling a rotation of the P_A_ keto group, the minimum distance to the Tyr OH of L-Y181 would be ~3.8 Å indicating only a very weak potential hydrogen bonding interaction (Fig. 4). Replacing Tyr M-210 with Phe has no effect on the orientation of the ring I keto group of P_B_. The native Tyr M-210 is apparently not hydrogen bonded to the ring I keto group of P_B_ (18) and no hydrogen bonding partner for this keto group is visible in the S221 RC structure. The equivalent of His L-168 near P_A_ is Phe M-197 near P_B_, which does not offer the option of establishing a hydrogen bond (Fig. 4 *C* and *D*). The ring-edge distance between B_A_ and Phe upon the M-Y210F substitution is ~4.3 Å. The OH dipole of Tyr L-181 is added near B_B_ upon the L-F181Y substitution, which can modulate the redox potential of B_B_ (12, 38). It should also be noted that at RT the barrier for rotation of the keto group is expected to be low and therefore in the absence of other stabilizing factors it is likely that a mixture of both orientations is present. Interestingly, a recent high-resolution RT structure for a different mutant (M-F197H) (39) reported the same orientation for only the keto group of P_A_ while all other BChl and BPhe were modeled with a keto group in the alternative orientation compared to the 2J8C and our structure.

### H_B_ and H_A_ Region.

**Fig. 5.**
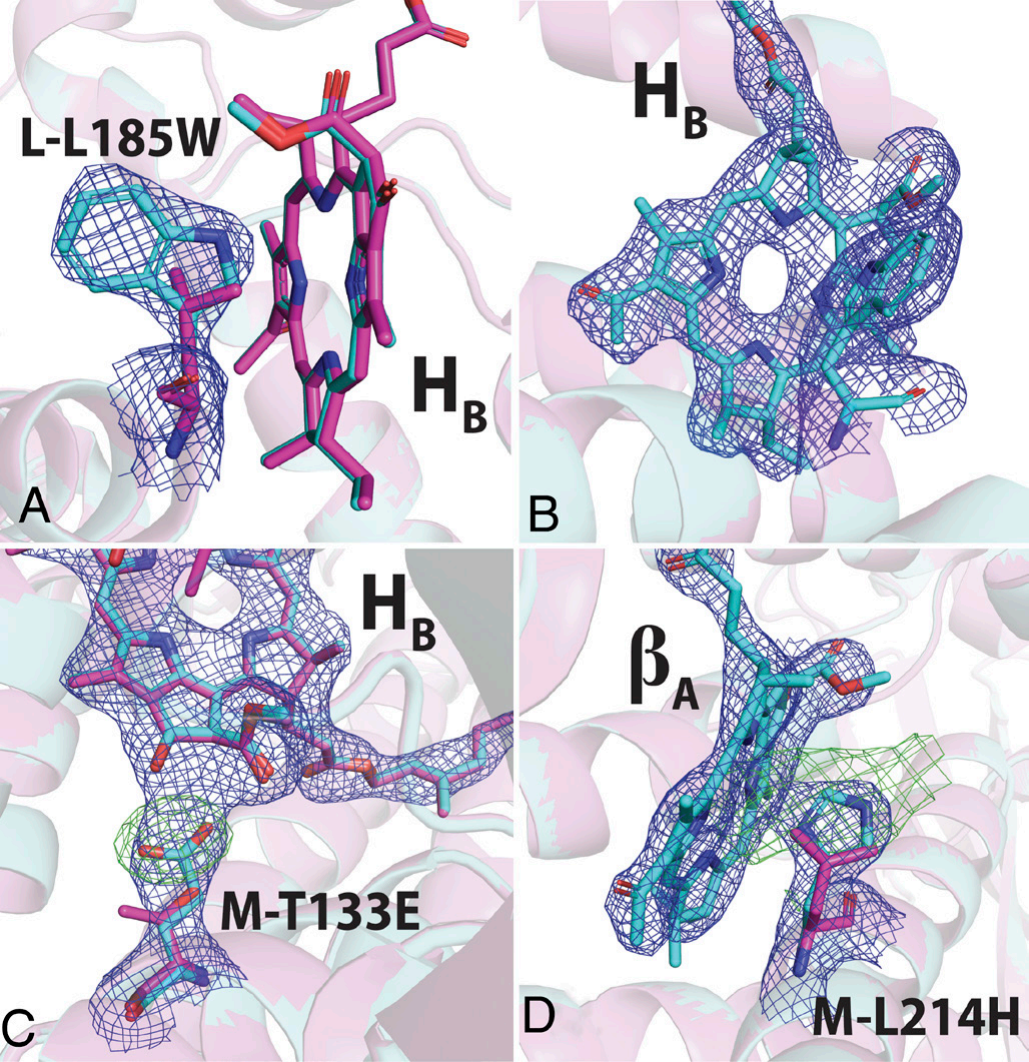
Changes around the positions of H_A_ and H_B_. Structures of the S221 variant RC (cyan) with the WT RC [PDB ID: 2J8C; (magenta)] are superimposed in the vicinity of the BPhs. (*A*) 2F_o_-F_c_ electron density contoured to 1.5σ (blue mesh) showing modeled position of L-W185 and BPh H_B_. In the S221 structure, the position of H_B_ is unchanged and the tryptophan substitution adopts a T-stacking conformation. (*B*) The lack of density in the interior of H_B_ indicates it has retained its BPh character. (*C* and *D*) 2F_o_-F_c_ electron density (blue mesh) contoured to 1.5σ, F_o_-F_c_ difference electron density (green mesh) contoured to 3.0σ for the H_B_ (*C*) and β_A_ (*D*) regions of the S221 RC. Amino acid substitutions are clearly visible in the density maps. The green difference electron density peak in the center of H_A_/β_A_ clearly shows that the BPh in the S221 RC has been converted to a BChl (β_A_), with the central Mg, by the M-L214H substitution.

The S221 structure shows that substitution of tryptophan for the leucine at L-185 results in an unexpected orientation of this residue, as seen in the F_o_-F_c_ electron density difference maps and 2mF_o_-DF_c_ electron density maps (Figs. 2*C* and 5*A*). One might expect a planar, face-to-face π–π interaction between the large aromatic group of tryptophan and the neighboring plane of the BPh ring (H_B_). However, an edge-to-face “T-stacking” orientation is observed with the five-membered ring of the tryptophan nearly perpendicular to the plane of the bacteriochlorin ring. The C_δ1_ (distal carbon adjacent to N_ε_ of the five-carbon ring of the tryptophan side chain) is pointed toward the N_δ_ in ring III of H_B_ at a distance of 3.1 Å. The 2mF_o_-DF_c_ and F_o_-F_c_ electron density maps confirm this result. To better understand the reason for the unexpected position of the tryptophan side chain, other orientations of the side chain were explored. While, in principle, other arrangements leading to a face-to-face π–π interaction with about 4 Å plane–plane distance between H_B_ and Trp L-185 are possible, they always lead to structural constraints. Clashes are found with Leu L-189 or the phytyl chain of B_B_ (*SI Appendix*, Fig. S2), indicating that the energy gain from forming a planar vs. a T-shaped π–π interaction is not likely to provide enough driving force for the needed displacement of other side chains/cofactors in the structure. This forced cofactor-side chain conformation may also be indirect evidence that incorporation of H_B_/B_B_ occurs concomitant with—rather than subsequent to—the protein folding process.

A large void in the 2mF_o_-DF_c_ electron density of H_B_ confirms that this cofactor remains BPh (i.e., has not been replaced with the Mg-containing analog BChl) (Fig. 5*B*). The substitution of M-T133 with a glutamic acid directly influences H_B_ by creating hydrogen bonding interactions with the ring V keto group and the adjacent carbonyl ester with distances of 2.7 and 2.8 Å, respectively. These interactions are seen in the electron density maps of this region (Fig. 5*C*). The interaction with the ring V keto group is similar to that of the Glu-L104 hydrogen bonded to H_A_ (M-T133E has distances of 2.7 and 2.8 Å with the ring V keto and the O1B ester groups of H_B_ whereas these distances are 2.6 and 3.5 Å in the WT A branch). Electrostatic calculations find Glu-L104 to be always protonated and to make a hydrogen bond with the nearby ring V keto group of H_A_ making the cofactor easier to reduce (13, 38). The extra electron-withdrawing character may help make H_B_ easier to reduce and thereby increase the yield of ET along the B-branch pathway.

The pseudo-symmetry-related cofactor H_A_ has been dramatically altered in the S221 RC by the substitution of a histidine in the position of M-L214. Electron density maps indicate that the N_δ_ of the imidazole ring of the histidine substituted in this position is coordinated to an ion (presumably Mg^2+^) located in the center of the bacteriochlorin ring (Fig. 5*D*). The previous void in the center of bacteriochlorin is now completely occupied in the 2mF_o_-DF_c_ map with electron density indicative of replacing the two central protons in the BPh H_A_ of WT with Mg, allowing unambiguous assignment of the density to a BChl (denoted β_A_) as a result of the M-L214H substitution (Fig. 5). This finding agrees with a prior structure of the RC carrying the single M-L214H substitution (40), and with prior spectral data and pigment extractions on other mutant RCs that contain the M-L214H substitution (20–23). Compared to the native BPh H_A_, ET to the new BChl β_A_ cofactor is hindered because BChl is 150 to 300 meV harder to reduce than BPh (Table 1).

### Quinone Binding Pockets—Q_B_ Region.

**Fig. 6.**
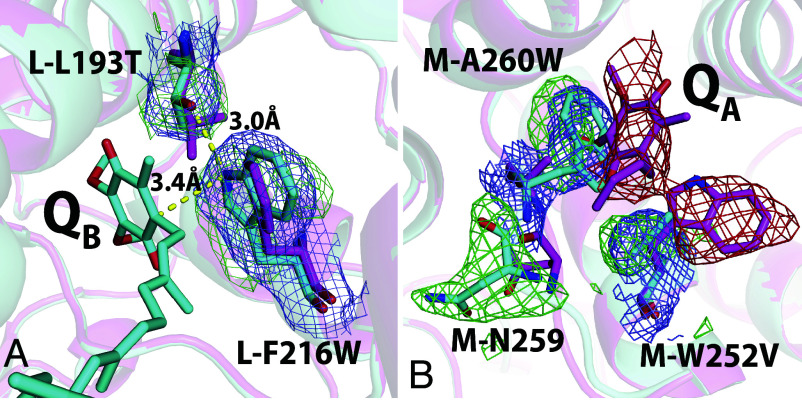
Changes around the Q_B_ and Q_A_ sites. Superimposition of amino acids in quinone binding pockets [S221 variant structure, cyan; WT structure (PDB ID: 2J8C), magenta] with 2F_o_-F_c_ electron density (blue mesh) and F_o_-F_c_ difference density (green and red mesh). (*A*) Q_B_ binding is stabilized by the π–π interactions with the indole N of the tryptophan substitution (L-F216W). (*B*) Q_A_ is excluded from the binding pocket due to steric clashes with the newly introduced Trp substitution at M-260, and the adjacent Asp259 undergoes an unexpected large conformational shift.

The pocket surrounding Q_B_ in the S221 RC has two site-directed substitutions (Table 1) aimed at increasing the occupancy of the quinone in this location. The amino acids Leu L-193 and Phe L-216 have been replaced with threonine and tryptophan, respectively. These residues are located on the distal side of Q_B_ relative to the central nonheme Fe and both are oriented parallel to Q_B_ (Fig. 6*A* and *SI Appendix*, Fig. S3*A*). These potentially increase the binding affinity of Q_B_ to this pocket, specifically in the proximal (as opposed to distal) position. The proximal position is near the Fe^2+^ while the distal position of Q_B_ has the quinone head group moved out into the channel for the quinone tail. The threonine and tryptophan are stabilized in these positions by each other through a hydrogen bond interaction with the O_γ_ of the Thr side chain and the N_ε_ of the Trp side chain. Although the Trp and Q_B_ are slightly offset, there is sufficient overlap for planar π–π interactions between the N_ε_ of Trp and the C_6_ of the Q_B_ ring at a distance of 3.4 Å, which may contribute to Q_B_ stabilization at this position (Fig. 6*A* and *SI Appendix*, Fig. S3*B*). Consistent with this stronger binding of Q_B_ is the observation that a ubiquinone can be modeled with 100% occupancy in the proximal position in the Q_B_ binding pocket, forming H-bonding interactions with His L-190 (2.7 Å), Ser L-223 (2.9 Å), and the backbone of Ile L-224 (3.1 Å) (*SI Appendix*, Fig. S3*B*). No indication of disorder or a second (distal) binding position is visible for Q_B_ in the S221 RC structure. In contrast, WT PBRC structures tend to have the quinone distributed between the distal and proximal positions (36, 41).

### Q_A_ Region.

Substitutions M-A260W and M-W252V, introduced to prevent Q_A_ binding at this site (Table 1), lead to dramatic alterations of the Q_A_ binding pocket and are the location for the largest changes between WT and S221 RCs (Fig. 6*B*). In the WT complex, Trp M-252 stabilizes Q_A_ occupancy via planar π–π interactions; with Val substituted at M-252, this interaction has been eliminated. Substituting Trp in place of Ala at M-260 introduces a large steric clash, excluding Q_A_ from this pocket. Clear F_o_-F_c_ difference electron density confirms this exclusion and the positions of the amino acid substitutions (Fig. 6*B*).

Unexpected additional structural changes are also seen in this region as consequences of the M-A260W and M-W252V substitutions. The most obvious change is seen for M-N259, the residue directly adjacent to M-W260 in the S221 RC structure. M-N259 has reoriented ~180° around the C_α_ backbone to a position distal to the Q_A_ pocket, shifts the C_α_ backbone 2.8 Å, and the side-chain amino group nitrogen is shifted by 6.5 Å (Fig. 6*B* and *SI Appendix*, Figs. S4 and S5). This large structural movement leads to a cascade of conformational changes in this region. First, the new position of M-N259 shifts the C_α_ backbone, the neighboring Phe at M-258 is also affected through a 1.5 Å movement of the C_α_ carbon, and the C_ζ_ carbon of the aromatic ring shifts by 1.8 Å (*SI Appendix*, Fig. S4). The movement of M-N259 also results in a repulsion of H-Q32 and H-Y40, with the C_δ_ of the H-Q32 side chain shifted by 2.3 Å and the hydroxyl oxygen of H-Y40 shifted by 1.3 Å (*SI Appendix*, Fig. S5).

There have been reports of an alternative binding site for ubiquinone in RC variants where the Q_A_ binding pocket was blocked (10). We investigated our electron density maps and found possible indication of a new molecule bound in a location between the original Q_A_ binding pocket and the region of the newly created BChl β_A_ in the vicinity of residues His M-214 and Met M-218. Modeling with an imidazole did not result in a good fit to the density. Instead, modeling the head group of a ubiquinone molecule resulted in a better fit with 75% occupancy and a slightly elevated B-factor compared to its surroundings. We labeled this potential distal partially occupied ubiquinone on the A branch as Q_A(d)_ (*SI Appendix*, Fig. S6), following a previously proposed nomenclature (10).

### Lipids.

At the present resolution the location of lipids is more challenging compared to cryogenic high-resolution structures and could also be complicated by higher mobility of lipid tails at room temperature compared to cryogenic measurements. Nevertheless we observed some clear changes between the WT and our S221 structures in the lipid structure that are likely not caused by limited resolution or only higher mobility but are results of the amino acid substitutions. While in the WT structure the lipid digalactosyldiacylglycerol (GGD) is present adjacent to the Q_A_ binding site, it is conspicuously absent in the structure of the S221 variant RC. The change might be caused by the movement of H-Q32, which now occupies part of the WT GGD binding site, and by H-Y40 moving closer to the Q_A_ binding pocket (*SI Appendix*, Fig. S5). Also absent from this structure is the phosphatidylcholine (PC1) near H_B_ and B_B_, with the phytyl tail of B_B_ now occupying this space and the phytyl tail of H_B_ shifting away from this pocket (*SI Appendix*, Fig. S7). An additional shift is observed in M-M256, which also moves to occupy the void where PC1 was located.

### Electrostatic Midpoint Potential Calculations.

In addition to structural studies described above, we calculated the midpoint potentials of the cofactors of the WT and the S221 variant using the MCCE program. These calculations were performed on the coordinates obtained from the RT structural data for the S221 RC presented above. The details of these calculations are described below. The ET reactions in photosynthesis occur by electron tunneling and the relative rates along the A and B branches will thus be affected by the distance between the cofactors and the free energy changes for each step. The A- and B-branch cofactor positions are very similar in the WT and S221 RCs as described above. Here, the free energy along the two branches is calculated using the MCCE program. The midpoint potentials of B_A_, B_B_, H_A_, and H_B_ and β_A_ in the S221 RC are compared with previously reported values (38) for the experimental structure of the WT RC from the carotenoidless strain R-26 (PDB ID: 1AIG) (41), as well as with the YF-swap (M-Y210F/L-F181Y) and YFβ_A_ (YF swap + M-L214H) mutant RCs prepared in silico.

**Fig. 7.**
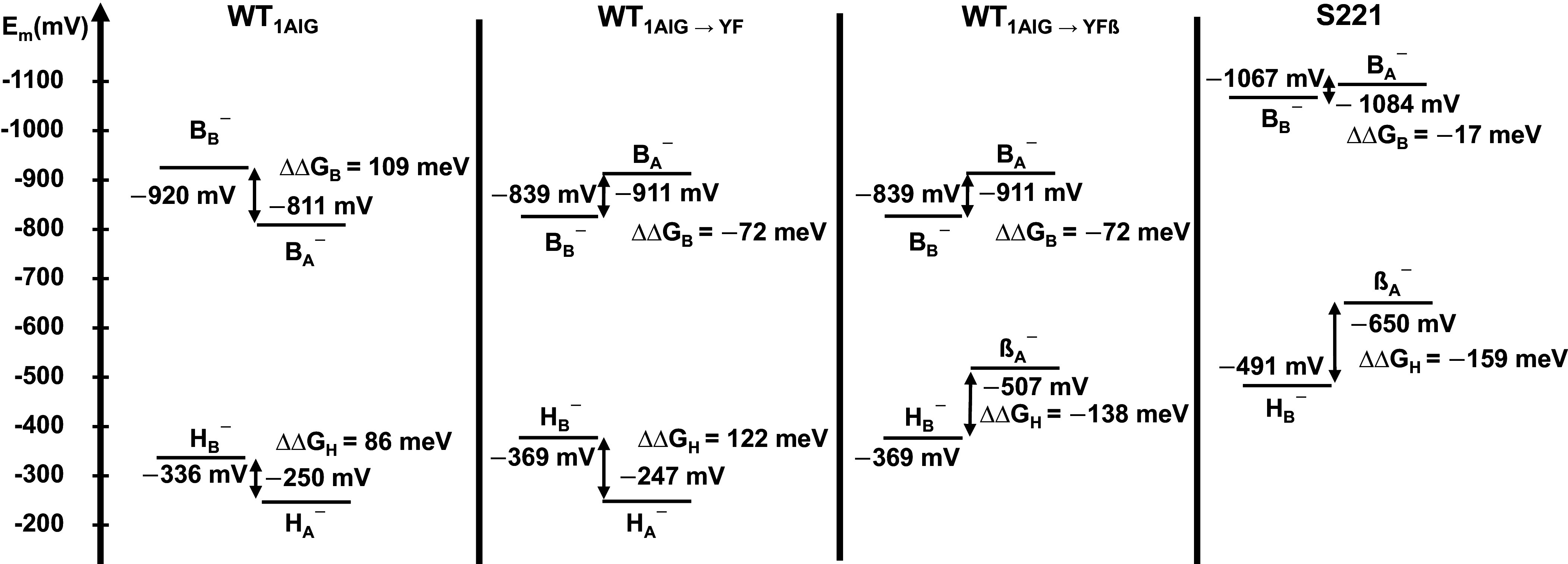
MCCE calculated midpoint potentials (E_m_) vs. standard hydrogen electrode for ET to cofactors along the symmetry-related A- and B branches for the WT RC structure (R-26 carotenoidless strain; PDB ID: 1AIG) and the structure of the S221 variant RC. Vertical arrows show ∆∆G between the A- and B-branch cofactors in meV. Positive ∆∆G values indicate that A-side reduction is favored relative to B-side reduction. TheYF and YFβ_A_ RC structures were prepared in silico from the WT structure (38). The estimated E_m_ change due to the substitution of a BChl in the H_A_ site is 260 mV, taking the same value found for ∆E_m_ between these cofactors in solution (24), resulting in a −507 mV E_m_ value for β_A_. E_m_ values are taken from Table 3.

MCCE calculates the shift in the E_m_ due to the electrostatic interactions of BChl or BPh in the ground state and in their reduced state due to the protein. In the description here, where the B side is the reference, more negative ΔΔGs(ΔG_A_-ΔG_B_) or more positive ΔE_m_s (E_m,A_-E_m,B_) favor A-side ET (Fig. 7). Table 3 shows that A-branch BChl reduction is favored in the WT RC by 80 mV and reduction of H_A_ is favored by 116 mV relative to the B-branch cofactors.

**Table 3.** Electrostatic midpoint potentials of each cofactor for the WT, YF, YFβ_A_, and S221 RC structures

Average (mV)	BChl_B_	BChl_A_	BPh_B_	BPh_A_	β_A_
E_m,sol_	−860	−860	−580	−580	*−860*
WT	−911	−831	−366	−250	*NA*
∆E_m,A-B_	*80*		*116*		*NA*
E_m_ YF and YFβ_A_	−839	−911	−369	−247	*−507*
∆E_m,A-B_	*−72*		*122*		*−138*
E_m_ S221	−1,067	−1,084	−491	x	*−650*
∆E_m,A-B_	*−17*		*−159*		

MCCE-calculated cofactor E_m_s. WT: R-26 (carotenoidless; PDB ID: 1AIG); YF: L-F181Y/M-Y210F swap prepared in silico from the WT structure within MCCE by modifying only the OH and H atoms of Tyr M-210 and Phe L-181 (38). YFβ_A_: The in silico YF swap was coupled to substitution of a BChl in the position of BPh_A_, resulting in a cofactor with an E_m_ that is 260 mV more negative that disfavors its reduction. S221: E_m_ calculations were performed using the coordinates of the S221 RC structure (this study). The differences between A- and B-branch values are shown underlined, in italics. Positive ∆E_m,A-B_ values favor A-branch reduction. NA: not applicable.

Two of the substitutions in the S221 RC were explored previously (38). With the in silico introduction of the YF swap (Tyr at M-210 near B_A_ and Phe at L-181 near B_B_ in the WT RC that are reversed in the S221 variant), the E_m_ reduction changes to favor B_B_ by 72 mV while the reduction of H_A_ is still favored on the A side (Fig. 7). This occurs because on either branch, the Tyr OH group makes the adjacent BChl easier to reduce. Thus, the native Tyr M-210 favors reduction of B_A_ while introducing Tyr L-181 in the S221 variant RC favors reduction of B_B_. However, replacing the BPh in the H_A_ site with BChl (β_A_) makes the cofactor harder to reduce, so that in the in silico YFβ_A_ variant RC, MCCE calculates that reduction of the B-branch B_B_ and H_B_ are favored over the A-branch cofactors. It follows that introducing nine site-directed substitutions in the S221 RC structure results in B_B_ reduction to be favored over B_A_ reduction by 17 mV, while H_B_ reduction is favored over reduction of β_A_ by 159 mV (Fig. 7). Thus, while the trends are the same, the calculated ΔE_m_ favors B_B_ less in the experimental structure than that predicted using a structure mutated in silico. H_B_ is slightly more favored relative to β_A_ in the experimental structure where it is influenced by additional substitutions that were not considered in the earlier calculations.

The MCCE program can deconstruct the factors that contribute to the calculated E_m_ shift in the cofactor midpoint potential (*SI Appendix*, Table S1) (38). The primary contributions come from the interactions with the axial ligands to BChl, with the partial charges on nearby neutral cofactors, with charged and polar side chains, and with the amide dipoles of the protein backbone.

*SI Appendix*, Table S1 compares the contributions to the electrostatic interactions in the structures of the WT and S221 variant RCs. The only substitutions that modify the BChls directly are the YF swap (M-Y210F/L-F181Y), which makes B_A_ harder to reduce and B_B_ easier to reduce. The S221 variant contains the M-L214H substitution that results in H_A_ being replaced by β_A_. The M-T133E substitution adds a Glu to form a hydrogen bond with the ring V keto group of H_B_ to mirror the role of Glu L-104 as a hydrogen bond donor to H_A_. MCCE predicts that Glu M-133 near H_B_ and Glu L-104 near β_A_ in the S221 RC, and Glu L-104 near H_A_ in the WT RC will be protonated and will make the adjacent cofactor (H_B_, β_A_, and H_A_) easier to reduce by ≈60 mV.

L-R103 and M-R132 are in homologous positions on the A- and B branches and are retained in the S221 variant RC. Both Arg residues favor reduction of their nearby cofactors more so in the variant than in the WT RC structure. Overall, the two Arg residues favor A-branch ET in the S221 RC structure relative to what is found for the WT RC structure. The nonheme iron sits on the symmetry axis between the two quinones. The Fe^2+^ stabilizes H_B_ less in the S221 RC than it does in the WT RC. Small changes in the overall structure lead to the backbone dipoles being less favorable for reduction of β_A_ in the S221 RC than H_A_ in the WT RC, while the solvation penalty for reduction of this β_A_ cofactor in the S221 RC is smaller than reduction of H_A_ in the WT RC structure, overall favoring β_A_ reduction (*SI Appendix*, Table S1). The contribution of changes in the effects of native amino acids shows the importance of doing calculations on an experimental structure of a variant RC, rather than making assumptions from a structure in which substitutions are made in silico.

## Discussion

### Background and Rationale.

The XFEL-based structural studies of the *C. sphaeroides* mutant RC (“S221”) presented here provide an important advancement in decades-long studies to elucidate the factors responsible for unidirectional ET via the A-branch cofactors in the native RC. Generating mutants to direct electrons “the wrong way” to the normally inactive B branch in high yield has been semirational but strategic. The S221 mutant RC is notable for several reasons: 1) ET from P* to the A-side cofactors is substantially detuned (10% primary A-side ET yield); 2) quinone Q_A_ is displaced so that any formation of P^+^Q_B_^−^ must occur by B-side charge separation; 3) the 68% yield of P^+^H_B_^−^ is among the highest observed, being similar to the yield in related variant RCs that lack substitutions near QB (11); 4) the 95% yield of ET from H_B_^−^ to Q_B_, resulting in a 65% overall yield of P* → P^+^Q_B_^−^ solely via B-side transmembrane charge separation, is the highest achieved to date (5).

The electron density map for the S221 variant RC is of sufficient quality to effectively sequence the peptides and confirm the amino acid substitutions (Fig. 2). Despite nine substitutions (Fig. 1 and Table 1), the positions and structure (e.g., extent of planarity) of the six bacteriochlorin cofactors are unchanged from the WT RC structure and the same is generally true of the nearby amino acids (with the exception of side chains of the substituted residues) (Figs. 2–5). This consistent layout of the bacteriochlorin cofactors emphasizes that subtle changes in the nature of side chain properties and orientations play large roles in cofactor energetics and redox properties to detune the ET steps on the A path and activate ET on the B path. In the S221 RC, the yield of ET from P* to the A-side cofactors (to produce P^+^β_A_^−^) has been reduced to 10% and the yield of B-side ET to produce P^+^H_B_^−^ is 68%, with the remaining 22% of P* decaying to the ground state. The MCCE electrostatic calculations show that reduction of A-side B_A_ and H_A_ is favored in the WT RC, while reduction of the B-branch cofactors B_B_ and H_B_ is favored in the S221 mutant RC (Fig. 7 and Table 3).

**Fig. 8.**
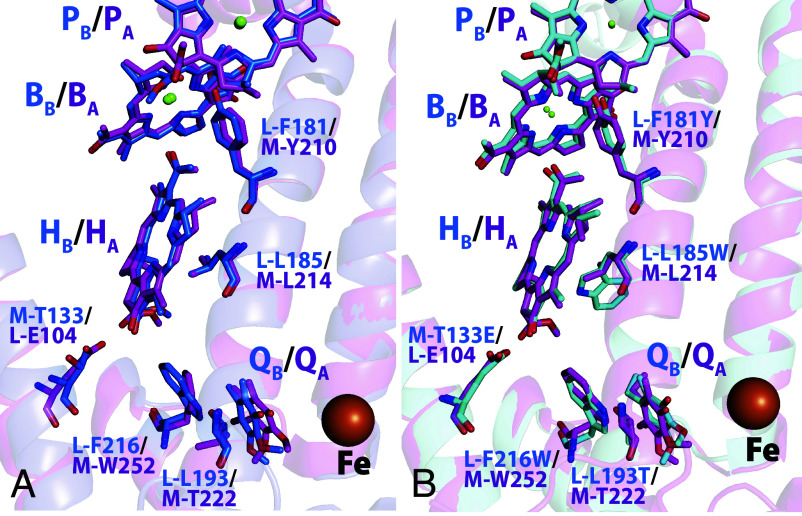
Superimposition of *A*- and *B*-branch cofactors and selected neighboring residues in the RC. (*A*) Overlay of the B branch (blue) and A branch (magenta) of the WT RC (PDB ID: 2J8C). Differences in side chains are visible, especially for the Q_A_ vs. Q_B_ binding pocket. (*B*) Superimposition of the B branch of the S221 variant RC (cyan) with the A branch of the WT RC (magenta).

In contrast to previous structures of variant RCs, the S221 RC has extensive modifications of residues surrounding the B-branch cofactors in addition to substitutions nearby A-branch cofactors. Direct superimposition of the A- and B-side cofactor branches of the WT RC (Fig. 8*A*) highlights the symmetry of both the cofactors and helices associated with them on the two sides. There are notable conserved asymmetries between the residues associated with the active A-side ET pathway and those associated with the inactive B branch. The realization of the S221 mutant RC targeted many of these amino acid asymmetries for improvement of B-side steps of both primary (P* → P^+^B_B_^−^ → P^+^H_B_^−^) and secondary (P^+^H_B_^−^ → P^+^Q_B_^−^) ET while disabling A-side ET steps. The strategy profited from the results of earlier site-directed mutagenesis in RCs of both *Rhodobacter* (*R.*) *capsulatus* and *C. sphaeroides*, saturation mutagenesis, in which all native amino acids were incorporated at a site (including L-181, L-185, and M-133) (11, 14, 15, 21, 42–44), and a Trp-scanning survey of the region between H_B_ and Q_B_ (including L-185 and L-216) to identify substitutions that increased the efficiency of this long-distance ET step (5). An important consideration in pursuing subsequent variants with additional amino acid substitutions was the structural stability of mutant RC complexes, as reflected by expression yield (*SI Appendix*, Fig. S1).

In summary, a variety of strategies was explored to realize the high yield of transmembrane charge separation achieved in the stable S221 mutant RC whose structure is reported herein. As shown in Fig. 8*B*, several of the conserved amino acid asymmetries that differentiate the two pathways in the WT RC have been eliminated or reversed in the S221 mutant RC (see also *SI Appendix*, Fig. S8 for comparison of the A- and B branches in the S221 RC). With the exception of the newly introduced Trp L-185, which is related by the twofold axis to Leu M-214 in the WT RC, the other B-side substitutions in the S221 RC result in a pathway that resembles the native A-side pathway in several key positions.

### Directionality of Electron Transfer.

A significant observation from the structure of the S221 RC is that the positions of the six bacteriochlorin cofactors are unchanged, confirming that the primary effect of the substitutions is to tune the energetics of ET on the two branches (Table 1). It was also recently proposed that subtle structural changes in the orientation of the ring I keto group of the bacteriochlorin cofactors could influence the charge separation pathway due to changes in the local electrostatics (45). While we did not observe any deviations in our structure from the previous high-resolution WT structure in terms of the orientation of the ring I keto groups (possibly due to limited resolution), a recent high-resolution RT structure for a different mutant (M-F197H, pdb 7P2C) (39) reported different keto group orientations for five out of the six bacteriochlorins, possibly indicating more flexibility of these groups at room temperature.

The tuning of the energetics of ET by individual amino acid substitutions is confirmed by the MCCE calculations and is manifest in the high yield of B-side ET. Four of the nine substitutions target detuning A-side ET (M-Y210F near B_A_, M-L214H near H_A_, and both M-W252V and M-A260W near Q_A_) and five target activating B-side ET (L-F181Y near B_B_, both M-T133E and L-L185W near H_B_, and both L-F216W and L-L193T near Q_B_). Some of these substitutions could potentially affect ET on both branches because they alter the P oxidation potential and, thereby, the free energies of all charge-separated states (which contain P^+^).

Although the individual M-Y210F and L-F181Y substitutions each alter the P/P^+^ potential, their effects are mirrored and juxtaposed so that the YF swap has little net effect on the P oxidation potential (42). The YF swap (Figs. 3 and 4) is predicted to contribute to the change in ET directionality because it increases the polar character of the binding pocket of B_B_ and makes the B_A_ binding pocket more hydrophobic. Previous calculations in the WT RC show that B_A_ is easiest to reduce (by up to ≈130 meV) if the dipole of the OH group of Tyr M-210 orients its positive end toward nearby B_A_ (13, 14, 38). The MCCE results presented here also suggest that the OH dipole of Tyr L-181 introduced in mutant RCs makes B_B_ easier to reduce, but by a lesser amount (*SI Appendix*, Table S1). In summary, the YF swap results in either a gain or loss of a polar functional group that promotes B-side rather than A-side ET.

The electron density map of the S221 RC indeed shows that BPh H_A_ is replaced by BChl β_A_ as a result of the M-L214H substitution (Fig. 5 *B* and *D*). On the A side, a general utility of the M-L214H substitution for studies of directionality is that it opens a Q_x_ spectral window for unambiguous assessment of P^+^H_B_^−^ formation and its yield (with only one BPh remaining in these RCs). Additionally, because BChl is much harder to reduce than BPh, the free energy of P^+^β_A_^−^ in the S221 RC is expected to be much higher than P^+^H_A_^−^ in the WT RC, contributing to detuning of A-side ET (Table 1 and Fig. 7).

The M-T133E and L-L185W substitutions make H_B_ easier to reduce. The Trp introduced at L185 in place of the native Leu contributes to π–π interactions with H_B_ in an interesting edge-to-face “T-stacking” orientation (Figs. 2*C* and 5*A*) to avoid clashes with nearby amino acids (*SI Appendix*, Fig. S2). The aromatic ring of Trp L185 is expected to stabilize the H_B_ anion in state P^+^H_B_^−^ (Table 1) just as Trp residues facilitate hole transfer in proteins (15). A hydrogen bond between the introduced Glu M-133 and the ring V keto group of H_B_ in the S221 RC mirrors the situation on the A branch in the WT RC where Glu L-104 provides a similar hydrogen bond to H_A_. Mutant RCs containing the L-E104L and M-T133E substitutions show the expected directions of shifts of the Q_x_ absorption bands of H_A_ and H_B_, respectively, with extents that depend on the other residues nearby (11, 21). The S221 RC structure shows that Glu M-133 is 2.7 and 2.8 Å from the ring V keto and O1B ester of H_B_, respectively whereas the corresponding distances between the native Glu L-104 and H_A_ in WT are 2.6 and 3.5 Å. Note that these are slightly changed in the S221 RC with 2.7 and 3.0 Å for the corresponding distances between Glu L-104 and β_A_, likely due to a slightly different orientation of the chlorin ring of β_A_ in the S221 RC compared to H_A_ in the WT (*SI Appendix*, Fig. S8). Whether these differences reflect a change in the overall strength of the hydrogen bonding interactions of H_A_ in the WT RC vs. H_B_ in the S221 RC is difficult to judge. Deciphering the relative impacts of the M-T133E substitution on B-side ET vs. the L-E104L substitution on A-side ET requires in-depth analysis of the relative contributions of two-step ET (P* → P^+^B_A/B_^−^ → P^+^H_A/B_^−^) vs. one-step superexchange ET (P* → P^+^H_A/B_^−^) on the two branches. In this regard, one must consider not only the effects of the substitutions on redox properties and the relative free energies of the states from the perspective of the Marcus rate vs. free energy relationship, but also that the relative energies contribute to the effective electronic couplings via the energy denominators for superexchange mixing evaluated using the potential energy surfaces for P*, P^+^B_A/B_^−^, and P^+^H_A/B_^−^.

### Impact on Primary Charge Separation.

The yield of P^+^H_B_^−^ in the simple M-YM210F/L-F181Y “YF-swap” mutant RC has not been measured. However, this yield must be larger than the yield of 4% for the M-L214H/M-W252V mutant RC in which BPh H_A_ is replaced by BChl β_A_ and Q_A_ is displaced and less than the 14% found for the RC that contains all four substitutions (11). These four substitutions are part of the S221 RC. Thus, the YF swap contributes moderately to redirecting electron flow from P* to the B-side cofactors instead of to the A-side cofactors. The additional key to obtaining the high (~68%) yield of P^+^H_B_^−^ in the S221 RC is stabilization of that state by making H_B_ easier to reduce (11). This is accomplished (to differing extents) by adding a hydrogen bond to H_B_ via the M-T133E substitution and/or charge stabilization via the L-L185W substitution. The structural consequences of these two substitutions are described above (Fig. 5) with the energetic consequences delineated by the MCCE calculations (Table 3, Fig. 7, and *SI Appendix*, Table S1).

As noted above, the structure of the S221 RC reinforces the view that the primary effect of the amino acid substitutions is to tune the energetics of ET, enhancing ET from P* to the B-side cofactors, and diminishing ET from P* to A-side cofactors, both of which compete with internal conversion of P* to the ground state. These yields for the S221 RC are 68%, 10%, and 22%, respectively. Further redox tuning is achieved in a set of mutant RCs related to S221 that have the same substitutions near the bacteriochlorin cofactors (and Q_A_ but not Q_B_) except that Arg rather than Trp replaces the native Leu at L185 near H_B_ (46). Having Arg rather than Trp at L185 boosts the yield of P^+^H_B_^−^ to ~90% (by making H_B_ easier to reduce), diminishes ET to the A side to ~0 (by making P harder to oxidize), and reduces internal conversion to ~10%. Arg at L185 compromises ET from H_B_ to Q_B_, which is not an issue with Trp at L185 in the S221 RC wherein that step has a near quantitative (95%) yield. Substitutions at L185 and at most other positions in the S221 RC and related mutant RCs are not expected to significantly alter the charge distribution in P* or potential differences in electronic couplings between the two macrocycles of P and the A- vs. B-side cofactors, all of which have been shown (from studies of heterodimer mutant RCs where one or the other bacteriochlorophyll of P is replaced with bacteriopheophytin) to make much smaller contributions than energetics to the directionality of ET (47).

### Factors Influencing Secondary ET and Quinone Binding/Function.

Considering the Q_A_ binding pocket first, a previous structure of a mutant RC (10) contained five amino acid substitutions—one on the B branch (L-F181Y) and the others on the A branch (M-G203D, M-Y210F, M-L214H, M-A260W). A 5% yield was reported for ET along the B branch to form P^+^Q_B_^−^. The modifications around the Q_A_ binding pocket share some similarities to the observations for the S221 RC. In both cases, Ala M-260 was replaced by a bulky Trp residue in order to prevent binding of ubiquinone in the Q_A_ pocket. The previous study (10) reported a reordering of the backbone region from M-258 to M-260 (*SI Appendix*, Fig. S4). In the S221 RC, the M-A260W substitution was coupled to replacement of Trp M-252 by a Val, further destabilizing the ubiquinone in the binding pocket. [Note that the M-W252V substitution alone is capable of displacing Q_A_ from the RC (26).] As a consequence of this complementary set of interferences, the S221 RC structure quantitatively lacks quinone on the A path in its normal binding pocket. While we observed a potential secondary Q_A_ binding position at lower occupancy (similar to the observed “Q_A(d)_” in ref. 10) it is located at a different position, close to the newly inserted His at position M-214 and a Met at M-218. In addition, the Cl^−^ that was seen previously (10) at the place of the original Q_A_ position is better modeled by a water in the S221 RC (*SI Appendix*, Fig. S6).

Turning to the Q_B_ binding pocket, the structural data presented here show that the combined substitutions in the S221 RC (notably L-F216W and L-L193T) result in Q_B_ fully occupying the site proximal to the nonheme Fe atom. This occupancy may be facilitated by a three-way interaction that involves a weak hydrogen bond between Thr L-193 and Trp L-216 that holds the indole ring of Trp L-216 in an orientation for maximal π–π interaction with Q_B_ (Fig. 6 and *SI Appendix*, Fig. S3). This arrangement of Trp L-216 and Q_B_, aided by Thr L-193, may increase electronic coupling between H_B_ and Q_B_. This combination of effects underlies the near-quantitative (~95%) yield of P^+^H_B_^−^ → P^+^Q_B_^−^ secondary ET in the S221 RC, requiring the presence of both Thr L-193 and Trp L-216 (5).

In comparison, other structures of WT RCs often show Q_B_ with varying degrees of occupancy, in the sites both proximal and distal to the Fe atom. When comparing the S221 RC structure with that of the previously published quintuple mutant (10), which had no substitutions in the vicinity of Q_B_, the differences in the Q_B_ pocket are obvious. While the structure of the quintuple mutant RC shows a ubiquinone molecule only in the distal position of the Q_B_ pocket, the structure of the S221 RC shows 100% of Q_B_ in the proximal position. Prior work has shown that binding of Q_B_ to the proximal site precedes its reduction by ET from either the A- or B-branch cofactors (43). Thus, the proximal positioning of Q_B_ observed in the S221 RC readies this cofactor for accepting an electron from H_B_ to form a semiquinone Q_B_^−^. The localization of the quinone exclusively in the active, proximal binding site is also likely to increase the yield of Q_B_^−^ as stable reduction of the quinone in the distal site is not observed (44, 48) since it is energetically much less favorable (49).

### Summary.

The structure of the *C. sphaeroides* mutant RC (“S221”) presented here provides a foundation for a deeper understanding of the principles responsible for unidirectional ET via the A-branch cofactors in WT RCs and directing electrons instead to the B branch. The structure shows that despite nine amino acid substitutions, the positions and macrocycle architectures of the six bacteriochlorin cofactors are unchanged from the WT RC structure; the same is generally true of the nearby amino acids (except for the substituted residues). The structure confirms previous hypotheses derived from functional characterization studies including i) interactions between targeted amino acids and the bacteriochlorin cofactors and ii) nuances of structure–function relationships that underpin binding of the terminal electron acceptor quinone Q_B_ in the proximal site—all key to obtaining a high yield of transmembrane charge separation via the B path. The structure also enables high-quality MCCE calculations, providing the coordinates to reliably gauge the cofactor-protein electrostatic interactions. The calculations, in turn, support the roles of the amino acid substitutions—as well as aggregated changes from the surroundings—in altering the redox properties of the cofactors and thereby the free energies of the charge-separated states. Manipulation of these energetics to detune the A-side cofactors and activate the B-side cofactors is central to redirecting electron flow from the native A branch of the WT RC to the B branch of the S221 mutant RC for rapid, high-yield transmembrane charge separation.

## Materials and Methods

A detailed description of the methods is given in *SI Appendix*. In short, expression yields of RCs from chemoheterotrophically grown cultures of carotenoid-containing *C. sphaeroides* were measured as described previously (50). RCs from dark-grown cultures of the S221 mutant strain were purified and crystallized as described previously (50). Serial femtosecond crystallography measurements were conducted on BL-3, EH2 at the SPring-8 Angstrom Compact Laser (SACLA) XFEL facility (51), using drop-on-tape (DOT) sample delivery (52). Data were processed using *cctbx.xfel* and DIALS (53, 54). Statistics for data processing and refinement can be found in Table 2. Molecular replacement, model building, and refinement used Coot (55) and *phenix.refine* (56). MCCE calculations use the same method and parameters described previously (38). Note that prior related MCCE calculations of the WT, YF, and YFβ_A_ RCs were referenced to two carotenoidless mutant bRCs, 1AIG and 2GNU and the carotenoid-containing WT structures, 2J8C (38). The same conclusions regarding the effects of the amino acids on redox properties and energetics were found using the three reference structures. MCCE, being a classical program, calculated the shift in the E_m_ in the protein from a value in solution. The references are −860 mV for BChl and −580 mV for BPh.

## Supplementary Material

Appendix 01 (PDF)

## Data Availability

Protein coordinates and structure factor data have been deposited in the Protein Data Bank [9zs8 https://doi.org/10.2210/pdb9ZS8/pdb (pdb_00009zs8) (57)].
