# Supplementary material for: High-yield charge separation along the alternative path in a photosynthetic reaction center: X-ray structure and electrostatic analysis
Source: Proc Natl Acad Sci U S A. 2026 Aug 10;123(33):e2609063123. doi: 10.1073/pnas.2609063123 (PMC13486545; doi:10.1073/pnas.2609063123)
Supplement: Supplementary file 1 [file pnas.2609063123.sapp.pdf]

PNAS

Supporting Information for

High-yield charge separation along the alternative path in a photosynthetic reaction center: X-ray structure and electrostatic analysis

Stephen M. Keable,[1†] Rongmei Judy Wei,[2,3] James C. Buhrmaster,[4] Stephen Hippleheuser,[4] Hiroki Makita,[1] Philipp S. Simon,[1], Gregory A. Tira,[4] Moritz Kretzschmar,[1,5] Isabel Bogacz,[1] Kaitlyn M. Faries,[7] Anthony Lan,[1] Isabela I. Nangca,[1] Miao Zhang,[1] Margaret D. Doyle,[1] Petko Chernev,[6] Asmit Bhowmick,[1] Daniel W. Paley,[1] Nicholas K. Sauter,[1] Aaron S. Brewster,[1] Kensuke Tono,[8,9] Shigeki Owada,[8,9] Junko Yano,[1] Vittal K. Yachandra,[1] M. R. Gunner,[2,3,10] Christine Kirmaier,[7] Dewey Holten,[7], Deborah K. Hanson,[4] Philip D. Laible,[4*] Jan F. Kern,[1*]

[1]Molecular Biophysics and Integrated Bioimaging Division, Lawrence Berkeley National Laboratory, Berkeley, CA 94720, USA
[2]Ph.D. Program in Chemistry, The Graduate Center, City University of New York, New York, NY 10016, USA
[3]Department of Physics, City College of New York, NY 10031, USA
[4]Biosciences Division, Argonne National Laboratory, Lemont, IL 60439, USA.
[5]Department of Biology, Humboldt-Universität zu Berlin, Berlin, Germany
[6]Molecular Biomimetics, Department of Chemistry — Ångström, Uppsala University, Uppsala, Sweden
[7]Department of Chemistry, Washington University, St. Louis, MO 63130, USA
[8]RIKEN SPring-8 Center, 1-1-1 Kouto, Sayo-cho, Sayo-gun, Hyogo 679-5148, Japan.
[9]Japan Synchrotron Radiation Research Institute, 1-1-1 Kouto, Sayo-cho, Sayo-gun, Hyogo 679-5198, Japan.
[10]Ph.D. Program in Physics, The Graduate Center of the City University of New York, New York, NY 10016, USA
†Present address: Neutron Scattering Division, Oak Ridge National Laboratory, Oak Ridge, TN 37830, USA

Email: laible@anl.gov or jfkern@lbl.gov

This PDF file includes:

Methods and References
Figures S1 to S8
Table S1

Methods

Reaction Center Sample Preparation

Expression yields from chemoheterotrophically-grown cultures (dark, semi-aerobic) of carotenoid-containing *C. sphaeroides* strains were estimated by purification of RCs from 2L of culture with Deriphat 160-C (1). For crystallization, RCs from dark-grown cultures of the S221 mutant strain were purified and crystallized as described previously (2). Crystals grew in approximately 5 days, yielding numerous small particles with a rod-shaped morphology and a maximum length of 100 µm along the long axis. Crystals were grown, harvested, and pooled by pipette just prior to data collection. All manipulations were carried out under low, red-light conditions, and crystal slurry solutions were kept suspended in gas tight syringes via a mechanical rotary apparatus before and during sample delivery to the XFEL beam.

Sample Delivery and Data Collection

Serial femtosecond crystallography measurements were conducted on BL-3, EH2 at the SACLA XFEL facility (3), Hyogo, Japan, under proposal 2022A8057. RC crystals were transported to the XFEL beam using a custom built drop-on-tape sample delivery system (4). Low light conditions were maintained in the XFEL beam hutch to prevent photoreduction of the samples during measurement. A focusing acoustic transducer was used to deposit 4 nL droplets of crystal slurry onto a moving Kapton tape running at a speed of 300 mm/sec in a sealed helium environment as described previously (4). For visualization of the setup, the standard diodes were changed to diodes that emit at 1040 nm that were triggered in stroboscopic mode to limit light exposure of the sample. Data were recorded on a Rayonix MX300-HS detector at a rate of 30 Hz in 4 by 4 binning and with a distance of 192 mm. The X-ray beam energy was 10 keV (1.24 Å), beam size was 3.1 µm by 2.2 µm (using Be lenses for focusing), a power of 780 µJ (power density of ~110 $\mu J/\mu m^2$), and a pulse duration of <10 fs.

Data processing, model building and map calculation

Data were processed using *cctbx.xfel* and DIALS (5, 6). Briefly, the *cctbx.xfel* GUI (7) was run on the SACLA computing cluster, with notable processing parameters including a detector gain of 0.46, searching for at most 3 lattices per image, and performing initial merging with *cctbx.xfel.merge* (5) without a reference model for scaling (just averaging the integrated intensities). The averaged data were then used as a scaling reference in a 'bootstrap' approach, similar to the method in *PRIME* (8). Structure factor uncertainties were derived from the ev11 model (9).
Statistics for data processing and refinement can be found in Supplementary Table 1. PDB ID: 2J8C was used as a phasing model for molecular replacement with Phaser-MR in the Phenix suite of programs (10). Model building was carried out iteratively using Coot (11) and *phenix.refine* (10) until a structure with satisfactory statistics was constructed. Secondary restraints were not needed due to the presence of all ligands intrinsically deposited in the Phenix database.

MCCE calculations

The calculations reported here use the same method and parameters described previously (12). The coordinates from the WT crystal structure of the carotenoidless *C. sphaeroides* R-26 PBRC (PDB ID: 1AIG)(13) and of the S221 variant RC were used. Note that prior related MCCE calculations of the WT, YF and YFβ_A RCs were referenced to two carotenoidless mutant bRCs, 1AIG and 2GNU and the carotenoid-containing WT structures, 2J8C (12). The same conclusions regarding the effects of the amino acids throughout the protein on redox properties and energetics (with relatively small differences in detail) were found using the three reference structures. Furthermore, it has been shown experimentally for an early mutant RC that ET rates and yields from P* to H_B are basically the same whether or not the carotenoid is present (14).

Glycerol (GOL), lauryl dimethylamine-n-oxide (LDAO), phosphate (PO4), heptane-1,2,3-triol (HTO), 1,2-diacyl-sn-glycero-3-phosphocholine (PC1) and digalactosyldiacylglycerol (GGD) were removed from the structures. A 31.5 Å slab was added around the hydrophobic region with the IPECE program (15) to model the region of the membrane. All water molecules in the structures were removed and replaced with implicit solvent.

The calculations used the isosteric level of MCCE conformer generation that provides choices of polar proton positions, residue ionization states, and cofactor redox states. PARSE charges were used for amino acids (16). The dielectric constant of the protein and membrane is 2, while 80 is used for the surrounding water. Parameters for BChl, BPh, and quinone are taken from Ishikita (17). MCCE brings the system to equilibrium at pH 8 with varying solution E_h. One cofactor is allowed to titrate at a time with the others fixed in their neutral, ground states. The E_m within the protein is found at the calculated midpoint of the titration, which follows the Nernst equation. MCCE, being a classical program, calculated the shift in the E_m in the protein from a value in solution. The references are -860 mV for BChl and -580 mV for BPh. See (12) for a more detailed description.

References

1. P. D. Laible, *et al.*, Quinone reduction via secondary B-Branch electron transfer in mutant bacterial reaction centers. *Biochemistry* **42**, 1718–1730 (2003).
2. C. A. Kors, *et al.*, Effects of impurities on membrane-protein crystallization in different systems. *Acta Cryst D* **65**, 1062–1073 (2009).
3. K. Tono, T. Hara, M. Yabashi, H. Tanaka, Multiple-beamline operation of SACLA. *J Synchrotron Rad* **26**, 595–602 (2019).
4. F. D. Fuller, *et al.*, Drop-on-demand sample delivery for studying biocatalysts in action at X-ray free-electron lasers. *Nat. Methods* **14**, 443–449 (2017).
5. A. S. Brewster, *et al.*, *cctbx.xfel*: a suite for processing serial crystallographic data. [Preprint] (2025). Available at: https://www.biorxiv.org/content/10.1101/2025.05.04.652045v1 [Accessed 15 October 2025].
6. G. Winter, *et al.*, DIALS: implementation and evaluation of a new integration package. *Acta Cryst. D* **74**, 85–97 (2018).
7. A. S. Brewster, I. D. Young, A. Lyubimov, A. Bhowmick, N. K. Sauter, Processing serial crystallographic data from XFELs or synchrotrons using the *cctbx.xfel* GUI. *Computational Crystallography Newsletter* 22–39 (2019).
8. M. Uervirojnangkoorn, *et al.*, Enabling X-ray free electron laser crystallography for challenging biological systems from a limited number of crystals. *Elife* **4** (2015).
9. A. S. Brewster, *et al.*, SAD phasing of XFEL data depends critically on the error model. *Acta Crystallogr D Struct Biol* **75**, 959–968 (2019).
10. D. Liebschner, *et al.*, Macromolecular structure determination using X-rays, neutrons and electrons: recent developments in Phenix. *Acta Crystallogr. D* **75**, 861–877 (2019).
11. P. Emsley, B. Lohkamp, W. G. Scott, K. Cowtan, Features and development of Coot. *Acta Cryst. D* **66**, 486–501 (2010).
12. R. J. Wei, *et al.*, Why the electron chooses one of two symmetry-related paths in the type II bacterial photosynthetic reaction centers. *Phys. Chem. Chem. Phys.* **27**, 24816–24826 (2025).
13. M. H. B. Stowell, *et al.*, Light-induced structural changes in photosynthetic reaction center: Implications for mechanism of electron-proton transfer. *Science* **276**, 812–816 (1997).
14. C. Kirmaier, *et al.*, Comparison of M-Side Electron Transfer in *Rb. sphaeroides* and *Rb. capsulatus* Reaction Centers. *J. Phys. Chem. B* **106**, 1799–1808 (2002).
15. Y. Song, J. Mao, M. R. Gunner, Calculation of Proton Transfers in Bacteriorhodopsin bR and M Intermediates. *Biochemistry* **42**, 9875–9888 (2003).

16. D. Sitkoff, N. Ben-Tal, B. Honig, Calculation of alkane to water solvation free energies using continuum solvent models. *J. Phys. Chem.* **100**, 2744–2752 (1996).
17. K. Kawashima, H. Ishikita, Energetic insights into two electron transfer pathways in light-driven energy-converting enzymes. *Chem. Sci.* **9**, 4083–4092 (2018).

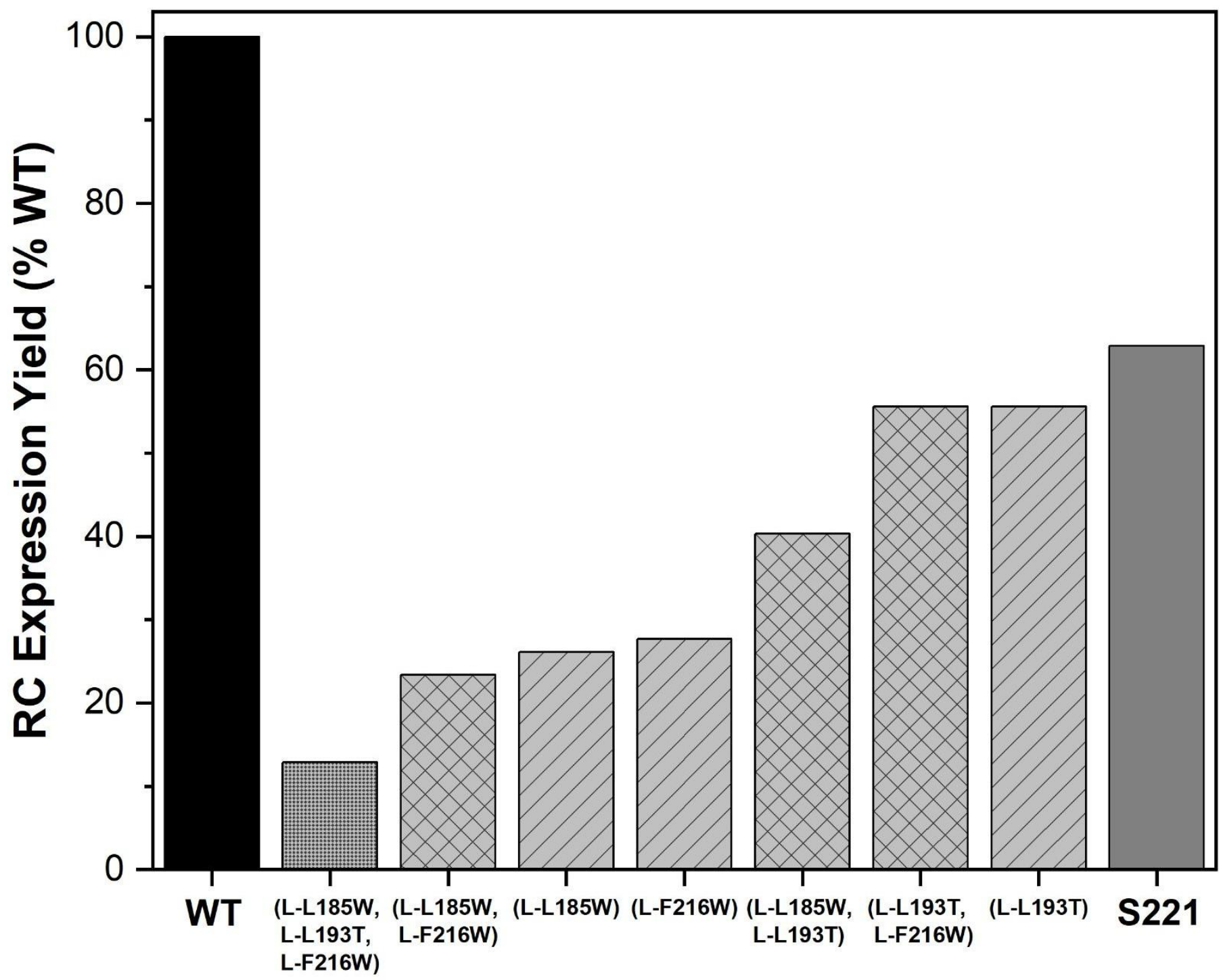

Fig. S1. Relative expression levels of mutant RCs. Yields of Deriphat-purified RCs, relative to the wild-type (WT), from the S221 mutant and predecessor strains that differ by carrying the WT residue at one (single hatch), two (crosshatch), or three (diamonds) of the nine sites of substitution in the S221 RC.

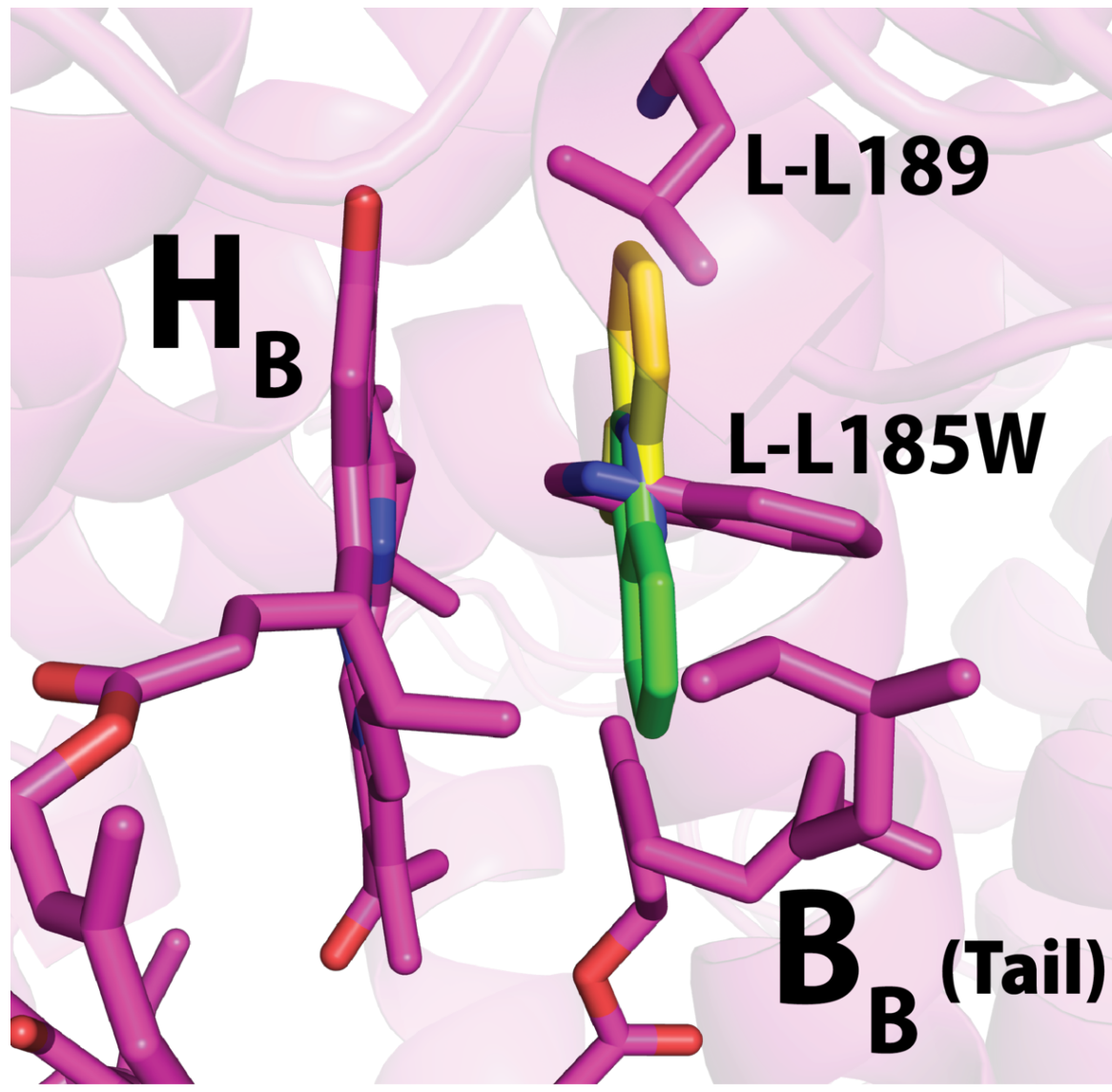

Fig. S2. Effects of forcing a face-to-face π-stacking interaction of Trp L-185 with H_B in the S221 RC structure. Trp L-185 is positioned in a tight pocket with little freedom to occupy any conformation other than a perpendicular, edge-to-face T-stacking orientation relative to H_B. The tryptophan will clash with the tail of B_B in one planar orientation (green), and Leu L-189 in the other planar π-stacking orientation (yellow).

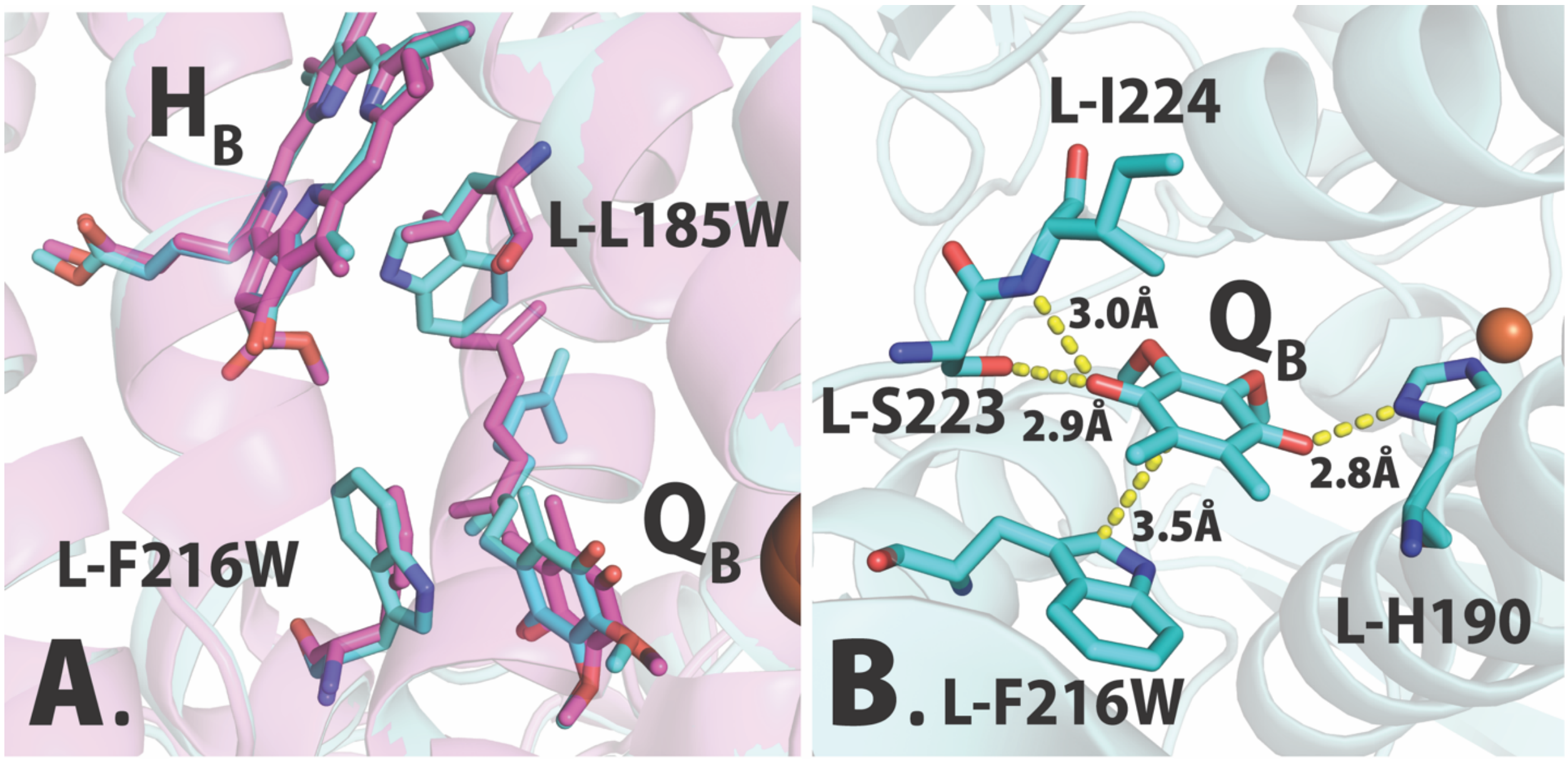

Fig. S3. The Q_B binding pocket and Tryptophan residues between H_B and Q_B. A. Superimposition of S221 variant RC structure (cyan) with WT RC structure (PDB ID: 2J8C). Tryptophan substitutions were used in a sampling strategy to increase π-electron coupling between H_B and Q_B to drive secondary ET along the B-branch pathway (*10*). **B.** Environment of Q_B in the S221 variant RC showing interactions with H-bonding partners and with Trp at position L-216.

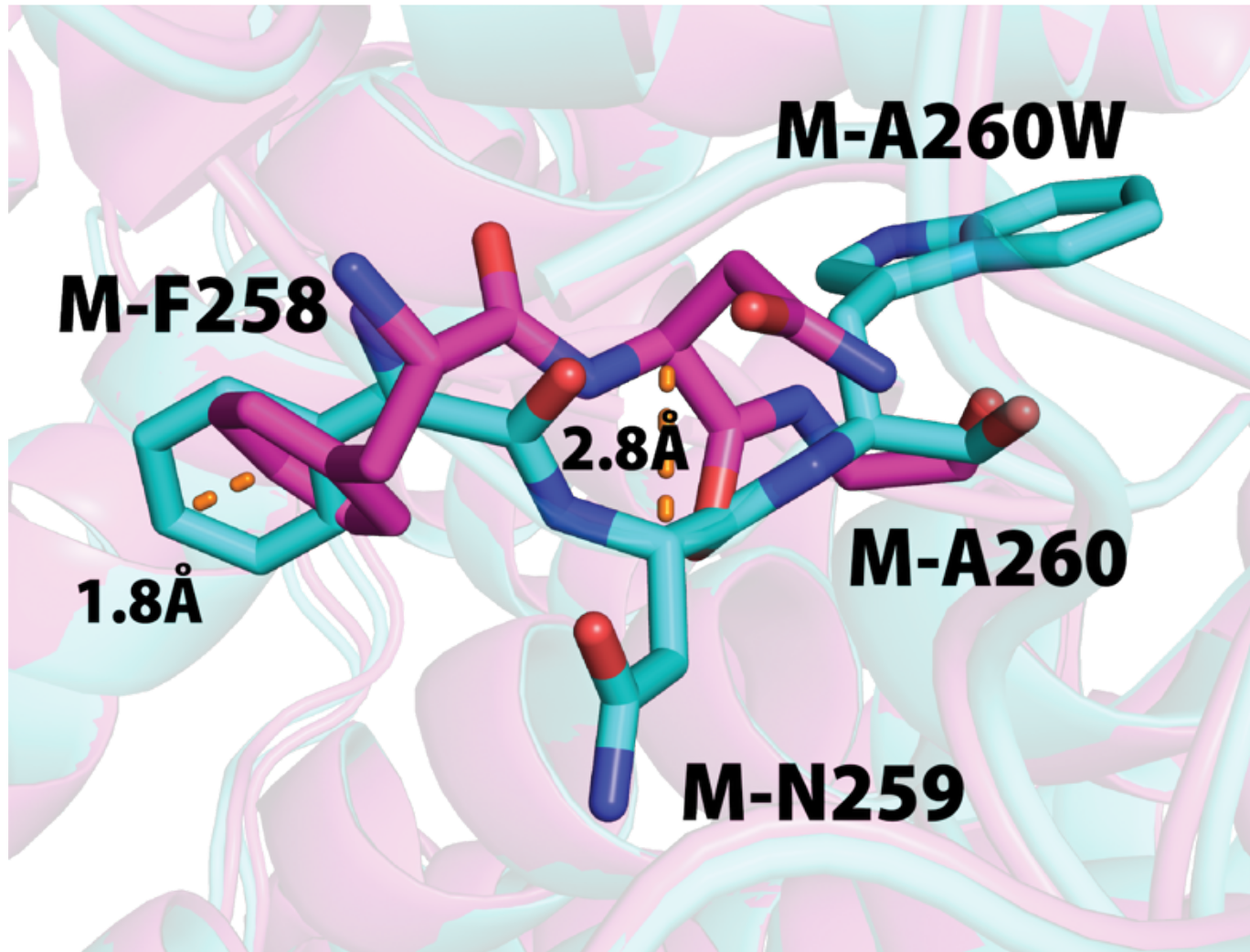

Fig. S4. Unpredicted structural consequences of the M-A260W substitution. The adjacent M-N259 residue undergoes a large conformational shift pivoting around the C_{α} backbone. M-F258 is also affected, sustaining a movement of the C_{α} and aromatic side chain. The S221 variant RC structure (cyan) is superimposed with the WT structure (magenta; PDB ID: 2J8C). Distances between nearest atoms of neighboring side chains are indicated by orange dashes.

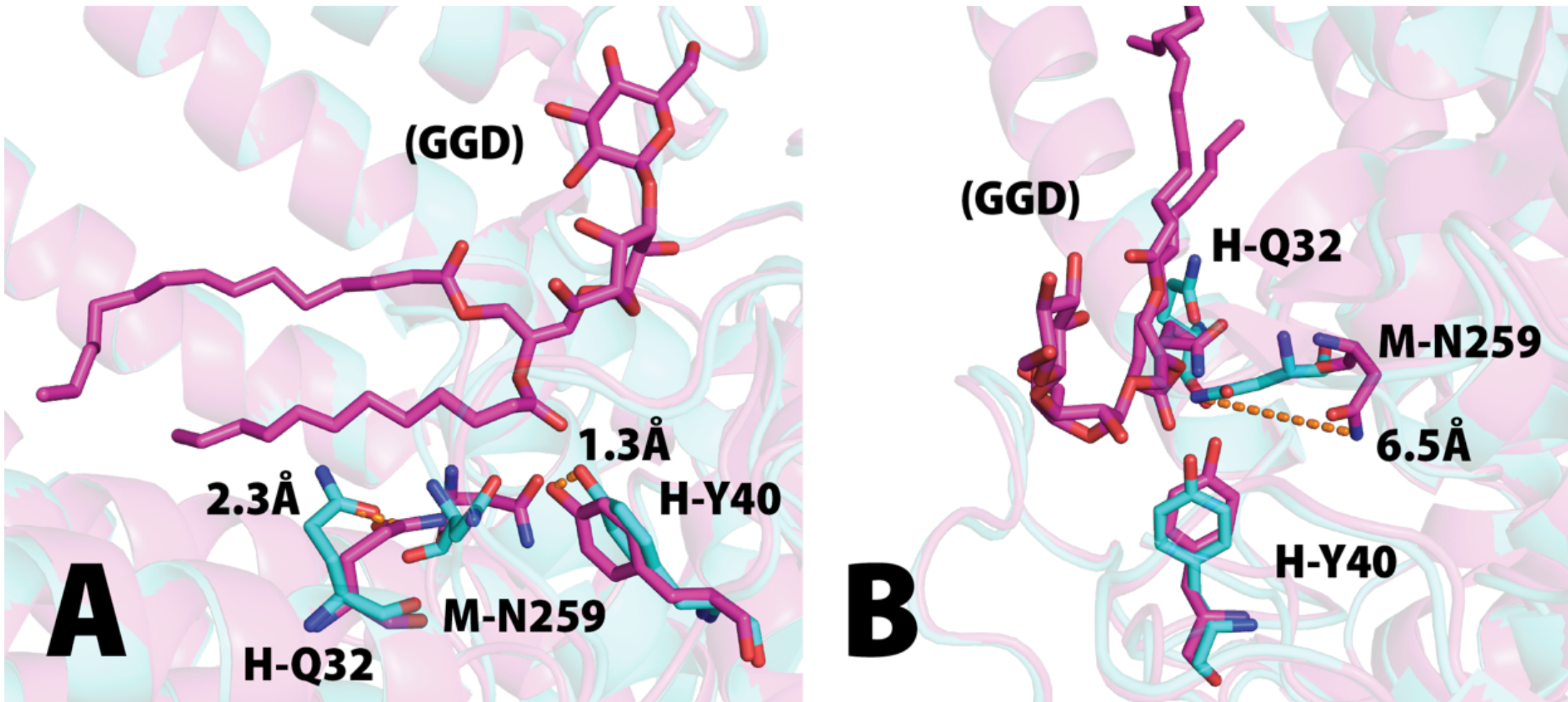

Fig. S5. The movement of M-N259 in the Q_A binding pocket also affects positions on the H chain. Structures of the S221 variant RC (cyan) and the WT RC (magenta; PDB ID: 2J8C) are superimposed. (**A**) H-Q32 and H-Y40 are repelled by the side chain of M-N259 undergoing R-group shifts of 2.3 Å and 1.3 Å, respectively. (**B**) A 180° view of this region highlighting the large 6.5 Å shift of the M-N259 side chain. Digalactosyldiacylglycerol (GGD) is absent in the S221 variant RC structure. Distances between nearest atoms of neighboring side chains are indicated by orange dashes.

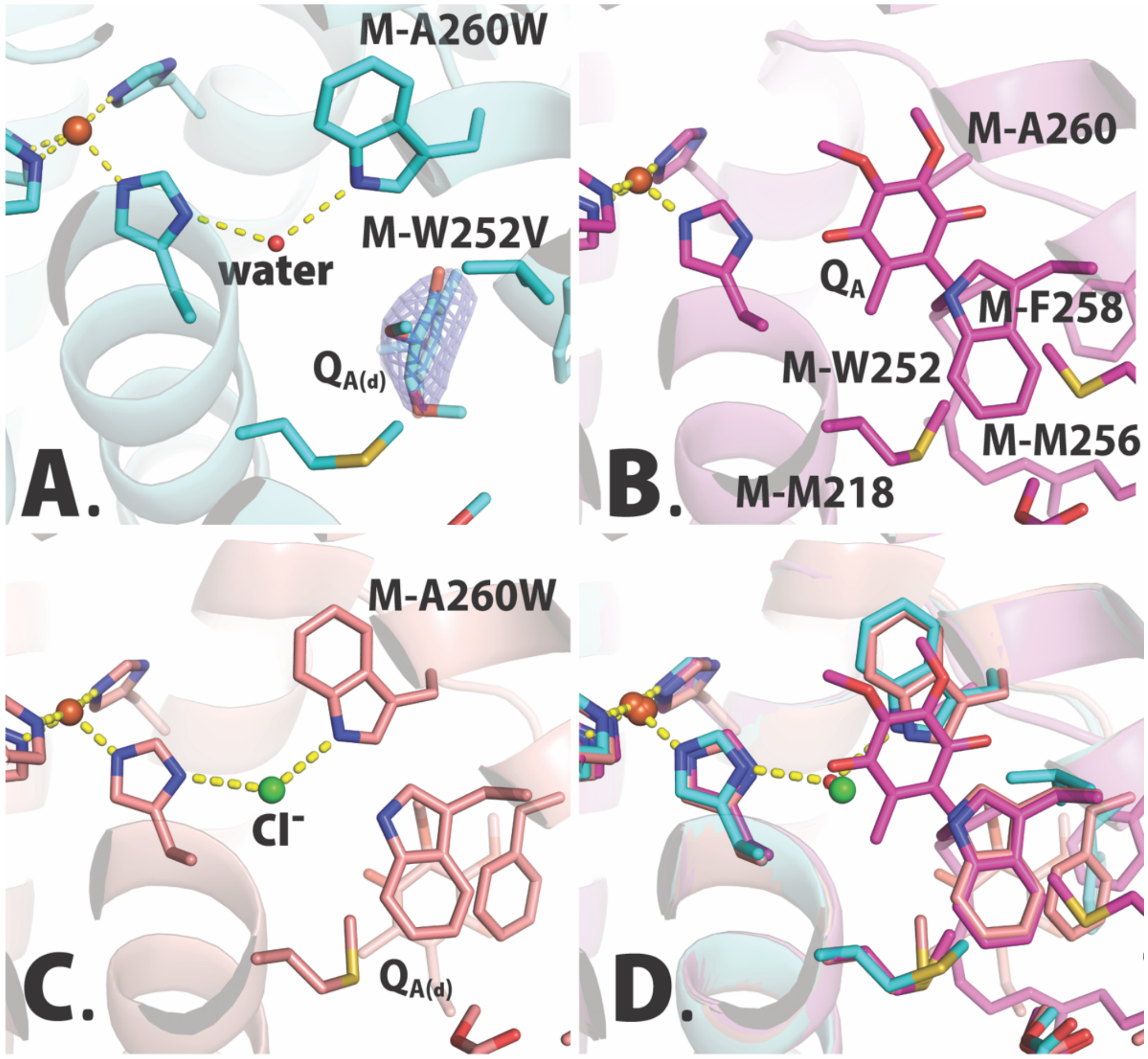

Fig. S6. Detail of changes in the Q_A binding pocket. (**A**) The region of the Q_A binding pocket in the S221 RC structure (cyan, 2Fo-Fc density for the distal quinone, $Q_{A(d)}$ is shown as mesh at 1.5 σ), (**B**) the WT RC (PDB ID: 2J8C; magenta) and (**C**) of a quintuple mutant RC (PDB ID: 1YF6 (*9*); salmon) and a superimposition (**D**) of all three structures. A Cl^- ion bound in the place of Q_A reported in the quintuple mutant RC (*9*) was not observed in the S221 RC structure. A secondary binding site for ubiquinone, labeled "$Q_{A(d)}$" was observed in the S221 RC structure, albeit at a different position compared to the one reported in the quintuple mutant. Distances between nearest atoms of neighboring side chains and the water molecule, and the Fe and Cl^- atoms are indicated by orange dashes.

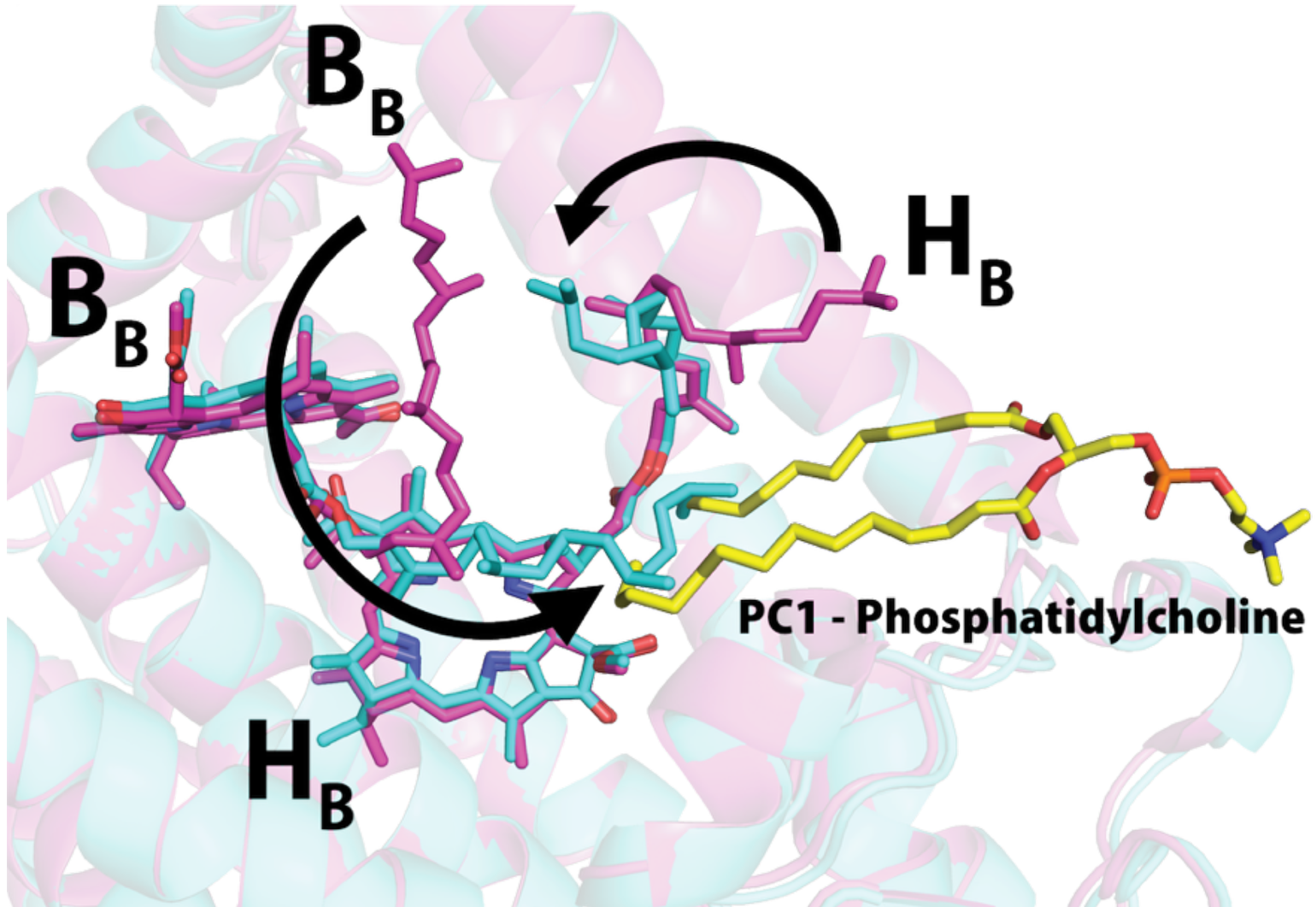

Fig. S7. Changes in positions of a lipid molecule and phytyl tails of B-branch cofactors of the S221 variant RC. Superimposition of the S221 variant RC structure (cyan) and the WT structure (magenta; PDB ID: 2J8C). Phosphatidylcholine (PC1) is not observed in the structure of the S221 RC. The redox-active cofactors H_B and B_B sustain structural rearrangements of the phytyl tails, with the tail of H_B occupying some of the region previously populated by the PC1 molecule.

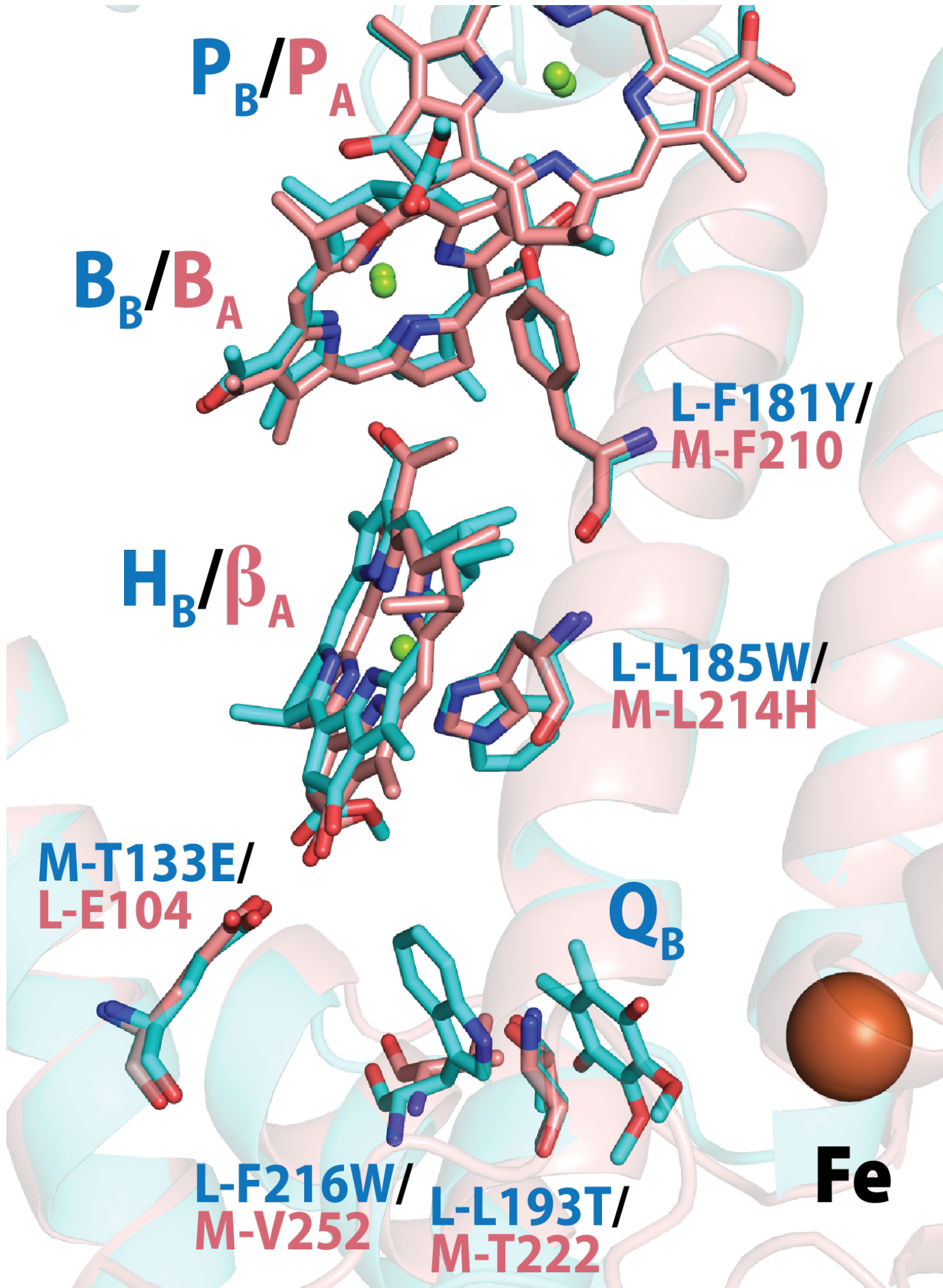

Fig. S8. Superimposition of the A-branch (salmon) and B-branch (cyan) of the S221 RC; view is similar to those shown in Figure 8 in the main text.

Table S1.1. ΔE_m calculated in the three *C. sphaeroides* RC crystal structures taken from (*21*) and the S221 RC structure (mV vs. SHE). ΔE_m values of WT and S221 for BChl and BPh of A and B branches. Comparison of S221 to WT is calculated by $\Delta E_{m,S221}$ -$\Delta E_{m,WT}$. The values for S221 A and B branches are listed in the last column, refer to $\Delta E_{m,S221}{}^{A}$ -$\Delta E_{m,S221}{}^{B}$.

**E_m (mV)**	**WT**	**S221**	**S221B-WT**	**E_m (mV)**	**WT**	**S221**	**S221-WT**	**S221_A-B**
$BChl_B$	-920	-1067	-147	$BChl_A$	-811	-1084	-273	-16
**E_m (mV)**	**WT**	**S221**	**S221-WT**	**E_m (mV)**	**WT**	**S221**	**S221A-WTA**	**S221_A-B**
BPh_B	-336	-491	-155	BPh_A	-250	-650	-400	-159

Table S1.2. Overview of contribution of each class of interaction to the free energy of reduction in the WT [taken from (*21*); PDB ID: 1AIG] and S221 RCs. Energy in meV. Negative ΔG contributions favor reduction of the cofactor; negative $\Delta\Delta G_{S221\text{-}WT}$ indicates that the cofactor in the S221 RC is easier to reduce than in the WT. Desolvation: loss of solvation energy of the charge moving from the reference solvent, water, to the position within the protein; Backbone: interaction with the amide backbone dipoles; Residue interaction divided into: Side chain + Fe^{2+}: Residues that shift the ΔG of reduction by more than ±29 meV (enumerated in SI table 1.3);; Background side chains: Summed interactions with residues that interact with the cofactor by less than ±29 meV; Cofactors+Ligand: interaction with the neutral neighboring cofactors and the axial ligands to $BChl_B$ and $BChl_A$ (His M-182 and His L-153, respectively). Residues_all: sums residue terms using mean field energy approximation.

SI Table 1.2a: $BChl_B$ (left) and $BChl_A$ (right)

ΔG° (meV)	WT	S221	$S221_B$-WT_B	ΔG° (meV)	WT	S221	$S221_A$-WT_A	S221_A-B
**Backbone**	-183	-160	23	**Backbone**	-192	-90	102	70
**Desolvation**	475	381	-94	**Desolvation**	459	116	-343	-265
**Cof+Lig_sum**	-188	-101	87	**Cof+Lig_sum**	-184	-143	41	-42
**Side chain+Fe^{2+}**	-356	-199	157	**Side chain+Fe^{2+}**	-530	-211	318	-12
**Background res_interaction**	11	-10	-21	**Background res_interaction**	126	118	-7	128
**Resi_all**	-345	-209	136	**Resi_all**	-404	-93	311	116

Table S1.2b. BPh_B (left) and BPh_A (right)

ΔG° (meV)	WT	S221	$S221_B$-WT_B	ΔG° (meV)	WT	S221	delta_S221-WT	S221_A-B
**Backbone**	-167	-164	3	**Backbone**	-140	-98	42	66
**Desolvation**	158	173	15	**Desolvation**	156	165	9	-8
**Cof+Lig_sum**	225	397	172	**Cof+Lig_sum**	224	353	129	-44
**Side chain+Fe^{2+}**	-228	-155	73	**Side chain+Fe^{2+}**	-288	-283	5	-128
**Background res_interaction**	74	-45	-119	**Background res_interaction**	-9	90	99	135

Resi_all	-154	-200	-46	Resi_all	-297	-193	104	7

Table S1.3. Effect of adjacent ground state cofactors on site reduction in the structures of the S221 and WT (PDB ID: 1AIG) RCs.
SI Table 1.3a: $BChl_B$ (left) and $BChl_A$ (right).

Cof_Lig Interactions (meV)	WT	S221	$S221_B$-WT_B	Cof_Lig Interactions (meV)	WT	S221	$S221_A$-WT_B	S221_A-B
P_B	-146	-76	70	P_A	-151	-97	54	-21
P_A	-57	-31	26	P_B	-71	-25	46	6
$BChl_A$	7	9	2	$BChl_B$	16	17	1	8
$BChl_B$	16	-9	-25	$BChl_A$	22	-37	-59	-27
BPh_A	-6	10	16	BPh_B	0	-3	-3	-13
BPh_B	NA	na	na	BPh_A	NA	na	na	na
Q_A	-1	na	na	Q_B	1	1	0	na
Q_B	-1	-4	-3	Q_A	-1	na	na	na
Sum_Cof+Lig	-188	-101	87	Sum_Cof+Lig	-184	-143	41	-42

Table S1.3b. BPh_B (left) and BPh_A (right)

Cof_Lig Interactions (meV)	WT	S221	$S221_B$-WT_B	Cof_Lig Interactions (meV)	WT	S221	$S221_A$-WT_A	S221_A-B
**His M-182**	134	206	72	**His L-153**	124	222	98	16
**P_B**	7	9	2	**P_A**	0	14	14	5
**P_A**	82	105	23	**P_B**	51	92	41	-13
**$BChl_A$**	25	22	-3	**$BChl_B$**	36	38	2	16
**$BChl_B$**	na	na		**$BChl_A$**	na	na	na	na
**BPh_A**	-5	19	24	**BPh_B**	2	-3	-5	-21
**BPh_B**	-17	36	53	**BPh_A**	11	-10	-21	-46
**Q_A**	-1	na	na	**Q_B**	1	0	-1	na
**Q_B**	0	-1	-1	**Q_A**	-1	na	na	na
**Sum_Cof+Lig**	225	397	172	**Sum_Cof+Lig**	224	353	129	-44

Table S1.4. Residue interactions that shift the free energy of cofactor reduction by more than ±29 meV. Each row has one residue with a strong interaction. The other residue in the row is the BLAST-aligned residue interacting with the cofactor on the other branch. Substituted residues shown in two rows that are boxed together. The top row is for the WT structure (PDB ID: 1AIG), bottom row in italics is for the S221 structure.

Table S1.4a. $BChl_B$ (left) and $BChl_A$ (right)

**Side chain+ Fe^{2+}(meV)**	**WT**	**S221**	**$S221_B$-WT_B**	**Side chain+ Fe^{2+}(meV)**	**WT**	**S221**	**$S221_A$-WT_A**	**S221_A-B**
**Phe L-181**	0	na	-63	**Tyr M-210**	-75	na	75	63
Tyr L-181*	*na*	*-63*		***Phe M-210****	*na*	*0*		
**Fe^{2+}**	-130	-113	17	**Fe^{2+}**	-151	-142	9	-29
**Glu M-234**	35	30	-5	**Glu M-234**	39	35	-4	5
**Arg M-164**	-59	-66	-7	**Arg L-135**	-60	-93	-33	-27
**Arg L-217**	-24	-22	2	**Arg M-253**	-31	-26	5	-4
**Arg L-231**	-35	-22	13	**Arg M-267**	-29	-26	3	-4
**Arg M-132**	-67	-6	61	**Arg L-103**	-89	-25	64	-19
**Leu M-135**	0	0	0	**Glu L-106**	41	7	-34	7
**Thr M-186**	-54	-51	3	**Val L-157**	0	0	0	51
**Glu L-212**	32	29	-3	**Arg M-241**	-8	-13	-5	-43
**Asp M-88**	32	67	35					
**Asp M-184**	42	-2	-44					
**Sum_Side chain+Fe^{2+}**	-228	-155	73	**Sum_Side chain+Fe^{2+}**	-288	-283	5	-128

Table S1.4b. BPh_B (left) and BPh_A (right).

Side chain+ Fe^{2+}(meV)	WT	S221	$S221_B$-WT_B	Side chain+ Fe^{2+}(meV)	WT	S221	$S221_A$-WT_A	S221_A-B
**Fe^{2+}**	-293	-233	60	**Fe^{2+}**	-310	-308	2	-75
**Leu L-185**	0	na	0	**Leu M-214**	0	na	197	197
Trp L-185*	*na*	*0*		***His M-214****	*na*	*197*		
**Thr M-133**	-6	na	-53	**Glu L-104**	-65	-34	31	25
Glu M-133*	*na*	*-59*						
**Phe L-216**	0	na	0	**Trp M-252**	0	na	0	0
Trp L-216*	*na*	*0*		***Val M-252****	*na*	*0*		
**Leu L-193**	0	na	-12	**Ala M-261**	0	na	4	16
Thr L-193*	*na*	*-12*		***Thr M-261****	*na*	*5*		
**GluL212**	84	75	-9	**Glu L-212**	40	37	-3	-38
**Glu M-234**	78	64	-14	**Glu M-234**	81	81	0	17
**Glu M-232**	34	28	-6	**Glu M-232**	37	33	-4	5
**Arg M-132**	-175	-26	149	**Arg L-103**	-165	-73	92	-48
**Arg M-164**	-45	-38	7	**Arg L-135**	-52	-44	8	-6
**Arg L-217**	-52	-40	12	**Arg M-253**	-58	-57	1	-17
**Arg L-231**	-52	-35	17	**Arg M-267**	-36	-41	-5	-7
**Arg M-267**	-48	-38	10	**Arg L-231**	-49	-28	21	11
**His M-182**	26	25	-1	**His L-153**	19	17	-2	-8
**Leu M-135**	0	0	0	**Glu L-106**	65	21	-44	21
**Ala M-139**	0	0	0	**Lys L-110**	-37	-16	21	-16
**Lys M-144**	-24	-19	5	**Tyr L-115**	-1	-2	-1	17
**Asp L-218**	55	42	-13	**Trp M-255**	14	1	-13	-41
**Asp L-213**	51	43	-8	**none**	na	na	na	na
**Glu H-173**	39	35	-4	**none**	na	na	na	na
**none**	na	na	na	**Arg M-247**	-35	-37	-2	na

**none**	na	na	na	**Glu L-6**	46	39	-7	na
**none**	na	na	na	**Arg M-228**	-27	-19	8	na
**Glu M-263**	25	26	1	**Glu M-263**	28	40	12	14
**Arg M-136**	-53	-26	27	**Arg L-10**	-25	-23	2	3
**none**	na	na	na	**Glu M-246**	20	34	14	na
**Arg M-241**	-7	-11	-4	**Arg M-241**	-16	-33	-17	-22
**Sum**	-356	-199	157	**Sum**	-530	-211	318	-12
